# Rotational Distributions and Imaging of Singlet O_2_ Following Spin-Forbidden Photodissociation of O_3_

**DOI:** 10.1021/acs.jpca.3c02736

**Published:** 2023-08-04

**Authors:** Megan N. Aardema, Megan Fast, Benjamen Meas, Simon W. North

**Affiliations:** Department of Chemistry, Texas A&M University, College Station, Texas 77842, United States

## Abstract

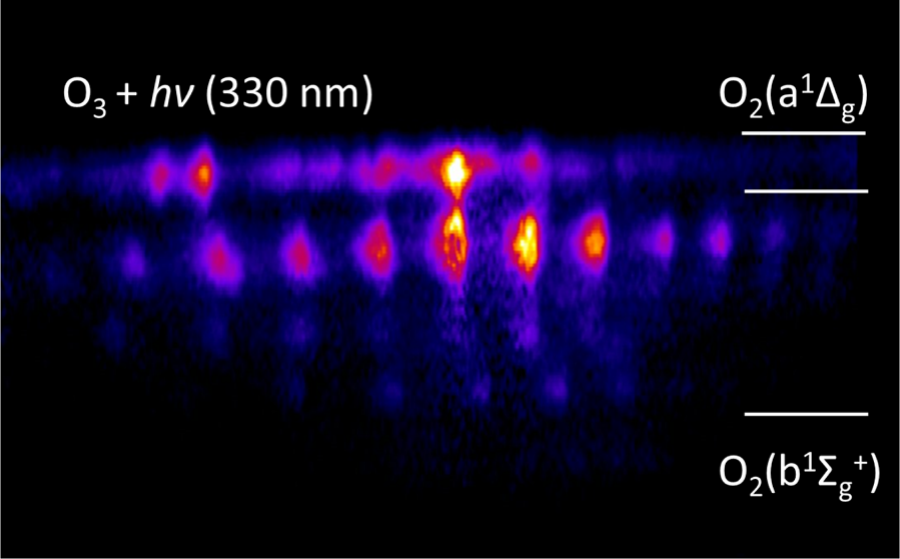

We report REMPI spectra and velocity-mapped ion images of the O_2_(*a*^1^Δ_*g*_) and (*b*^1^Σ_*g*_^+^) fragments arising from the spin-forbidden photodissociation of O_3_ near 320 and 330 nm. The O_2_(*a*^1^Δ_*g*_, v = 0) REMPI spectrum following a 320 nm dissociation shows enhanced peak intensity for the odd rotational states relative to the even states, which is the opposite of the trend observed by Gunthardt et al. (J. Chem. Phys.2019, 151, 22430231837678 10.1063/1.5131504) for spin-allowed dissociation at 266 nm but is consistent with the couplings between the B state and ^3^A′ and ^3^A″ states calculated by Grebenshchikov and Rosenwaks (J. Phys. Chem. A2010, 114, 9809–981920509638 10.1021/jp1028849). There are no significant differences between the ion image angular distributions of fragments in odd and even rotational states, which indicates a cold distribution of O_3_ and supports the explanation that the alternation in peak intensities results from a difference in the couplings. Quantitative analysis of the image angular distributions was limited due to the single laser polarization geometry accessible in one-color experiments. Radial distributions of the 320 nm images indicate a broad rotational distribution, evidenced in bimodal speed distributions with peaks corresponding to both high (*j* = 35–43) and low (*j* = 17–20) rotational states. The REMPI spectrum of O_2_(*a*^1^Δ_*g*_) near 330 nm was collected, and while quantitative population analysis is difficult because of the perturbed resonant state, the spectrum clearly supports a broad rotational distribution as well, consistent with the images collected at 320 nm. A 2D-REMPI spectrum was collected following dissociation of O_3_ near 330 nm, which showed evidence of contributions from O_2_ fragments in both the *a*^1^Δ_*g*_ and *b*^1^Σ_*g*_^+^ states. The rotational distribution for the O_2_(*b*^1^Σ_*g*_^+^, v = 0) product peaks at *j* = 32 and is narrower than that of the O_2_(*a*^1^Δ_*g*_) fragment, consistent with distributions reported by O’Keeffe et al. at longer dissociation wavelengths (J. Chem. Phys.**2002**, 117, 8705–8709). At smaller radii in the 2D-REMPI spectrum, there is additional signal assigned to v = 1–4 of O_2_(*b*^1^Σ_*g*_^+^), with rotational distributions similar to v = 0. The vibrational distribution of the O_2_(*b*^1^Σ_*g*_^+^) fragment peaks at v = 0, with populations monotonically decreasing with increasing vibrational state. Ion image angular distributions of the O_2_(*b*^1^Σ_*g*_^+^) fragment and the corresponding anisotropy parameters are also reported.

## 1 Introduction

The photodissociation of ozone in the Hartley and Huggins bands has been the focus of numerous experimental and theoretical studies due to its critical role in atmospheric processes.^1−37^ Absorption in both bands is due to transitions from ground state, O_3_(X^1^A′), to the B state (3^1^A′). In the Hartley band, excitation occurs to the continuum of the B state above the dissociation threshold, and the absorption spectrum is broad, peaking near 260 nm. There are two dissociation channels observed in the Hartley band: 90% of dissociations proceed diabatically on the B state, resulting in excited singlet products, O(^1^*D*) and O_2_(*a*^1^Δ_*g*_), while 10% cross from the B state to the repulsive R state, leading to ground-state triplet products, O(^3^*P*) and O_2_(*X*^3^Σ_*g*_^–^).^14−18^ In the Huggins band, excitation occurs to the bound region of the B state, resulting in a highly structured absorption spectrum with sharp peaks corresponding to vibrational transitions at cold temperatures.^2,38−43^ Both O(^1^*D*) and O_2_(*a*^1^Δ_*g*_) have been observed at dissociation wavelengths longer than 310 nm, despite the spin-allowed singlet channel being energetically inaccessible. While some of the singlet products originate from vibrationally excited parent molecules that have sufficient energy to dissociate via the spin-allowed singlet channel, there has been the production of O(^1^*D*) and O_2_(*a*^1^Δ_*g*_) at dissociation wavelengths longer than 310 nm, which is attributed to spin-forbidden dissociation processes based on the translational energy of the fragments.^3−5,24^ In the Hartley and Huggins bands, there are five energetically accessible dissociation channels,
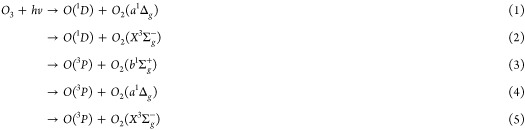
where channels 1 and 5 are spin-allowed and channels 2–4 are spin-forbidden following initial excitation to the B state. Studies of the O(^3^*P*) fragment following photodissociation in the Huggins band reported branching ratios of channels 3–5 to be approximately equal,^4,6^ and studies of the O(^1^*D*) fragment at long dissociation wavelengths have indicated a yield of 0.1.^7,17^ Thus, spin-forbidden processes are significant sources of excited O and O_2_ products in the Huggins band.

The spin-allowed channels 1 and 5 have been studied extensively following Hartley band dissociation both experimentally and theoretically. In the triplet channel (5), the O_2_(X^3^Σ_*g*_^–^) products are highly vibrationally and rotationally excited, and the vibrational distribution is strongly dependent on wavelength, with increasing vibrational excitation at shorter dissociation wavelengths.^8,9^ The spatial anisotropy (β) in the triplet channel varies with the rotational state as a result of bending motion at the time of dissociation, with positive β values at low rotational states and negative β values at high rotational states. In the singlet channel (1), the O_2_(*a*^1^Δ_*g*_) fragments are less vibrationally excited, with distributions peaking at v = 0 at all wavelengths.^8−10^ The rotational state distribution shifts to higher rotational states with higher-energy dissociations, and calculated distributions for this channel match experimental distributions.^8,19,30,31^ For all wavelengths, the singlet channel β is positive, increasing with longer dissociation wavelengths as a result of the restoring force in the bending potential, which more strongly affects fragments with slower recoil velocity.^8,31,32^

The rotational distribution of the singlet channel O_2_(*a*^1^Δ_*g*_) has been of particular interest because of the observed alternation of odd and even rotational states,^19,30^ which is attributed to a Λ-doublet propensity that leads to a preference for even rotational states.^11,30,33^ A nonrotating O_3_ molecule has A′ symmetry with respect to the molecular plane, so there is a preference to conserve symmetry and form the O_2_ A′ Λ-doublet, which must correspond to even rotational states due to symmetry restrictions. This alternation is highly temperature-dependent. There are three rotation axes in ozone: one axis is perpendicular to the molecular plane and two are in the molecular plane, and the final fragment angular momentum has components along all three axes. At higher temperatures, rotation about the two in-plane axes results in a rotation of the molecular plane and tilts the fragment O_2_ rotation plane relative to the initial plane. This breaks the symmetry restrictions and allows mixing of the A′ and A″ Λ-doublets. This results in a greater population of odd rotational states of O_2_. The angular distributions of the O_2_ ion images in odd and even rotational states also support the idea that odd j-state fragments originate from warmer parent distributions, with weaker correlations between the fragment velocity ***v*** and angular momentum ***j*** vectors as well as the transition dipole moment **μ** and ***j*** vectors in odd rotational states. The ***v*** and ***j*** vectors are expected to be perpendicular in the dissociation of a triatomic molecule, but greater out-of-plane rotation of the parent molecule decreases the angle between ***v*** and ***j***.

**Figure 1:**
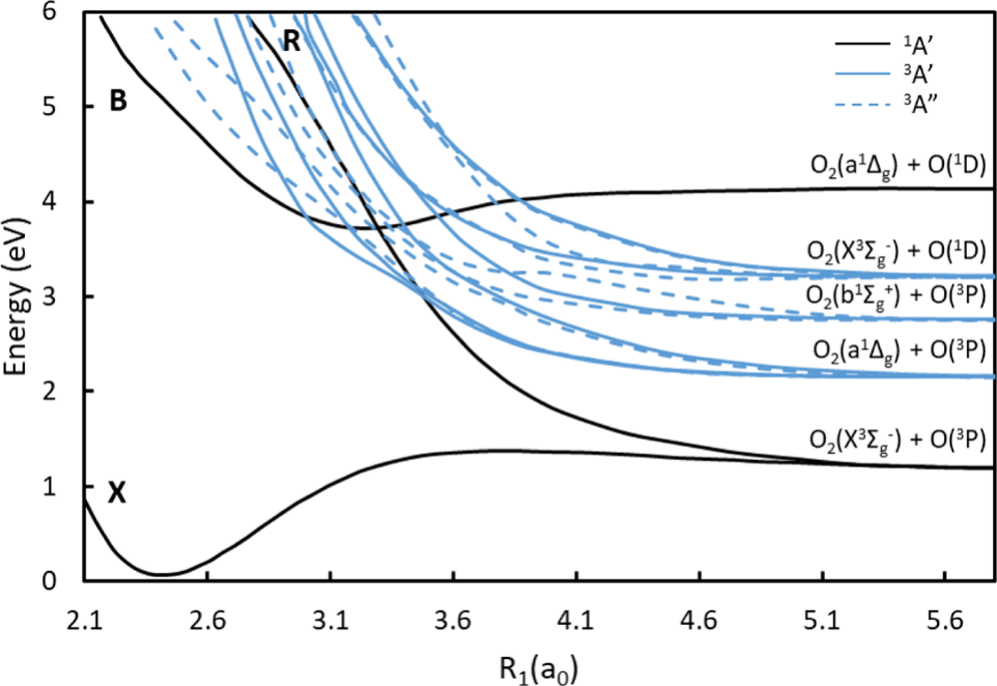
Potential energy curves calculated by Grebenshchikov and Rosenwaks.^12^ Solid black lines indicate ^1^A′ states, including the X, B, and R states. Solid and dashed blue lines indicate ^3^A′ and ^3^A″ states, respectively, that cross the B state and lead to spin-forbidden products.

There have been far fewer dynamics studies involving Huggins band dissociation of O_3_ below the energetic threshold for the spin-allowed singlet channel. Absorption in the Huggins band is much weaker than the Hartley band due to a large difference between the X and B equilibrium geometries and exhibits vibronic structure. Figure 1 shows potential energy curves calculated by Grebenshchikov and Rosenwaks for a fixed bond length of the second O–O bond (2.43 *a*_0_) and bond angle (117°).^12^ The X, B, and R states are all ^1^A′ and are shown in black. These three states account for all of the observed dynamics in Hartley band dissociations. In the Huggins band, initial excitation is to the bound region of the B state, but only triplet states correlate to spin-forbidden products. The triplet states that cross the B state and lead to spin-forbidden products are shown in Figure 1 in blue. Following initial excitation to the bound region of the B state, there are pathways involving both ^3^A′ states (solid lines) and ^3^A″ states (dashed lines) that cross the minimum of the B state and correlate with spin-forbidden products. The relative importance of interactions between the B state and the ^3^A′ and ^3^A″ states in spin-forbidden processes have not been well characterized, but Grebenshchikov and Rosenwaks calculated couplings between the B state and each of the ^3^A′ and ^3^A″ states for a single geometry.^12^

In addition to the measurement of the O(^3^*P*_0_) fragment translational energy suggesting equal branching between channels 3–5,^4^ REMPI spectra of the *b*^1^Σ_*g*_^+^ state were collected by O’Keeffe et al.^13,34^ The *b*^1^Σ_*g*_^+^ state REMPI was fit to obtain rotational state distributions, which O’Keeffe et al. reported peaked at *j* = 28 for a 344 nm dissociation and *j* = 34 for a 337.2 nm dissociation. More recently, Ulrich et al. collected ion images of O(^3^*P*_2_) following dissociation between 321 and 329 nm.^6^ The speed distributions of these images were fit to estimate the vibrational state distributions of each of the three channels, assuming a single rotational temperature for all three electronic states of O_2_. However, the unresolved nature of the derived speed distributions, due to the large number of possible coincident O_2_ fragment states, precludes quantitative analysis. Measuring the O_2_(*a*^1^Δ_*g*_) or O_2_(*b*^1^Σ_*g*_^+^) molecular fragments directly provides an opportunity to gain additional insight into the dissociation dynamics by resolving the rotational and vibrational distribution of the O_2_ fragment. Ulrich et al. also reported spatial anisotropy values for the O(^3^*P*_2_) images, with β ranging from −0.59 to 2.00 for individual features in the images assigned to O(^3^*P*_2_) formed with O_2_ in the X^3^Σ_*g*_^–^, *a*^1^Δ_*g*_, or *b*^1^Σ_*g*_^+^ states. Since the assignment of peaks in the radial distributions of these images is difficult, the wavelength dependence of β in each of the channels (3–5) is unclear. Images of the O_2_ fragment would complement the O(^3^*P*_2_) images, and obtaining β values from the O_2_ images would provide valuable information on individual dissociation channels and facilitate comparison to theory.

## 2 Experimental Section

The molecular beam/velocity map ion imaging apparatus has previously been described.^44,45^ The O_3_ molecular beam was generated by flowing He over O_3_ trapped on silica beads at −40 °C for a total pressure of 800 Torr, expanded through a General Valve series 9 pulse valve and collimated by a 1 mm conical skimmer. The molecular beam was intersected at 90° by a linearly polarized laser beam generated by frequency doubling the output of a dye laser (LAS LDL 2051) operating at 640–660 nm, pumped by a frequency-doubled Spectra Physics LAB 150–10 Nd:YAG laser. The wavelength of the laser was calibrated by optogalvanic spectroscopy, using a Mg–Ne or Cu–Ne hollow cathode lamp. The O_2_ fragments were probed using 2 + 1 Resonance-Enhanced Multiphoton Ionization (REMPI) via the *d*^1^Π_*g*_ state. The lower vibrational states (v = 0–3) of the *d*^1^Π_*g*_ state are perturbed by the nearby *II*^1^Π_*g*_ valence state and therefore are not ideal for extracting accurate rotational state distributions, but v ≥ 4 lie above the dissociation threshold of the *II*^1^Π_*g*_ state, are unperturbed, and therefore can be used to obtain rotational state information. The 2–0 band of the O_2_(*d*^1^Π_*g*_ ←← *a*^1^Δ_*g*_) transition at 320 nm was used for O_2_(*a*^1^Δ_*g*_) ion images and a REMPI spectrum for comparison to the 266 nm dissociation study of Gunthardt et al.^11^ A 2D-REMPI spectrum^46−49^ was collected near 330 nm in which images were obtained at each wavelength step and the reconstructed radial distributions were plotted as a function of wavelength. 330 nm corresponds to the 4–0 band of the O_2_(*d*^1^Π_*g*_ ←← *b*^1^Σ_*g*_^+^) transition and the 1–0 band of the O_2_(*d*^1^Π_*g*_ ←← *a*^1^Δ_*g*_) transition. Wavelengths near 330 nm were used to measure the rotational and vibrational state distributions of the O_2_(*b*^1^Σ_*g*_^+^) fragment, as well as to collect ion images. The rotational distribution of O_2_(*a*^1^Δ_*g*_, v = 0) at 330 nm is also discussed.

Following ionization, the O_2_ cations were accelerated by velocity mapping electrostatic lenses down a 50 cm field-free flight tube coaxial with the molecular beam and projected onto a position-sensitive detector composed of microchannel plates gated to selectively detect the mass of interest and a phosphor screen. The resulting images were collected by a CCD camera. For the 1D rotational spectra, a mask was used to block the low-velocity fragments and nonresonant signal in the O_2_(*d*^1^Π_*g*_, v = 2 ←← *a*^1^Δ_*g*_, v = 0) REMPI spectrum at 320 nm and the center spot in the O_2_(*d*^1^Π_*g*_, *v* = 1 ←← *a*^1^Δ_*g*_, v = 0) REMPI spectra at 330 nm, and the signal of interest was collected using a photomultiplier tube (PMT).

Typical laser energies were 4–5 mJ for the 320 nm 1D-REMPI, 6–8 mJ for the 330 nm 1D-REMPI, and 9–10 mJ for the 330 nm 2D-REMPI. All experiments were one-color experiments, using a vertically polarized laser (parallel to the imaging plane). The polarization of the laser was controlled with a photoelastic modulator and a Glan polarizer.

The beam temperature was estimated by NO calibration. A molecular beam of NO in He was used with seeding similar to that of a typical O_3_ beam, and the same pulse valve timing was used. NO was probed using the A ← X transition via 1 + 1 REMPI at 226 nm. The 226 nm laser had 50–100 μJ of power, and the ion optics were defocused to spread out the NO signal from the center of the image. The NO REMPI spectrum was fit with LIFBASE to obtain a beam temperature of 50 K.^50^ The spectrum and fit are shown in the Supporting Information.

## 3 Results and Discussion

### 3.1 Photodissociation near 320 nm

#### 3.1.1 Even–Odd Alternation in the O_2_(*a*^1^Δ_*g*_) Fragment

**Figure 2:**
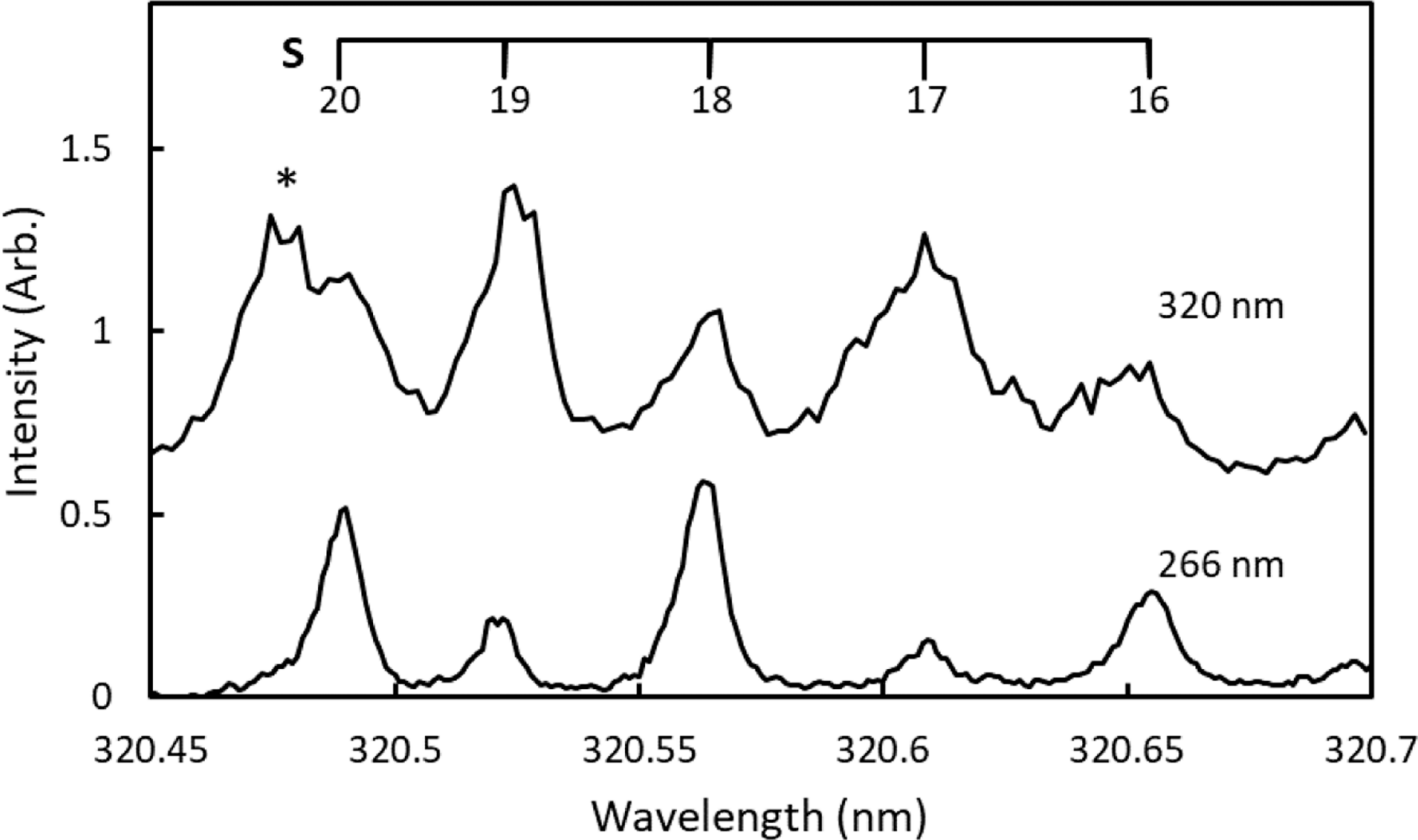
REMPI spectrum of the O_2_(*a*^1^Δ_*g*_) fragment following the dissociation of jet-cooled O_3_ probed via the O_2_(*d*^1^Π_*g*_, *v* = 2 ←← *a*^1^Δ_*g*_, v = 0) transition. The bottom spectrum was collected following dissociation at 266 nm^11^ and the top spectrum near 320 nm in a one-color experiment. S-branch transitions are indicated by the comb at the top, and the peak marked by an asterisk corresponds to the overlapping P-branch *j* = 42 transition.

For photodissociation at 320 nm, the spin-allowed dissociation channel producing O_2_(*a*^1^Δ_*g*_) and O(^1^*D*) is not energetically accessible from cold parent molecules. In one-color images of the O_2_(*a*^1^Δ_*g*_) fragments following 320 nm dissociation of O_3_, two regions of resonant signal were observed: a sharp outer ring at large radii and a broader ring at smaller radii (vide infra). The signal at smaller radii, corresponding to slower fragment velocities, is due to spin-allowed dissociation via channel 1 originating from vibrationally excited O_3_. The low-velocity signal was blocked with a mask and, therefore, does not contribute to the REMPI spectrum shown in Figure 2. The outer ring corresponds to spin-forbidden dissociation producing O_2_(*a*^1^Δ_*g*_) and O(^3^*P*) (channel 4) based on analysis of the derived speed distribution.

Figure 2 shows a comparison of the O_2_(*d*^1^Π_*g*_, v = 2 ←← a^1^Δ_*g*_, v = 0) REMPI spectrum following 320 nm dissociation (top) in a one-color experiment and the spectrum reported by Gunthardt et al.^11^ following a 266 nm dissociation (bottom). The peaks correspond to the S-branch transitions of rotational states *j* = 16–20. There is an overlap with the P-branch *j* = 42 transition in the 320 nm spectrum (indicated by an asterisk). Hartley band dissociation of O_3_ at 266 nm with a rotational temperature of 100 K shows significant suppression of the odd rotational states of O_2_, which results from a Λ-doublet propensity in which the A′ Λ-doublet is primarily formed to conserve the A′ symmetry of the parent O_3_. Due to symmetry restrictions, the A′ Λ-doublet leads to even rotational states of O_2_ and the A″ Λ-doublet leads to odd rotational states; therefore, the preference for the A′ Λ-doublet leads to a preference for even rotational states of O_2_(*a*^1^Δ_*g*_). The Λ-doublet propensity and the resulting alternation between the even and odd rotational states are highly temperature-dependent since the out-of-plane rotation of the parent O_3_ mixes the Λ-doublets. As a result, there is a greater population of odd rotational states and less alternation between the even and odd rotational states following dissociation of a warmer parent rotational distribution.

While the peaks corresponding to odd rotational states are highly suppressed following a 266 nm dissociation, the odd peaks are clearly enhanced following a 320 nm dissociation, despite the colder beam temperature of 50 K. Although quantitative rotational state populations cannot be obtained from the 2–0 band of the *d*^1^Π_*g*_ ←← *a*^1^Δ_*g*_ transition because of the highly perturbed nature of the low vibrational states of the O_2_(*d*^1^Π_*g*_) state, a comparison of the two spectra shown in Figure 2 suggests a fundamental difference between dissociation at 320 and 266 nm. We believe this is due to greater coupling between the B state and repulsive ^3^A″ states relative to the coupling between the B state and ^3^A′ states leading to O(^3^*P*) and O_2_(*a*^1^Δ_*g*_) products.^12,35^ Grebenshchikov and Rosenwaks calculated spin–orbit matrix elements between the B state and the repulsive ^3^A′ and ^3^A″ states producing spin-forbidden products for a single geometry (*R*_1_ = 2.43 *a*_0_, *R*_2_ = 3.20 *a*_0_, α = 117°) and found stronger coupling between the B state and the ^3^A″ repulsive states which would lead to odd rotational states of O_2_, consistent with experiment.^12^ The relative probabilities of transitions from the B state to ^3^A″ states compared to transitions from the B state to ^3^A′ states can be estimated with a 1D Landau–Zener model,
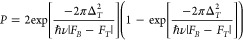
6where P is the probability of transition, Δ_*T*_ is the spin–orbit matrix element between the B and triplet states, ν is the relative velocity, and *F*_*B*_ and *F*_*T*_ are the slopes of the B and triplet state potentials at the crossing point, respectively. The probability was calculated for each triplet state with its corresponding spin–orbit matrix element from Grebenshchikov and Rosenwaks, reflecting the coupling of the triplet state to the B state. Summing the probabilities of transition to each of the individual ^3^A′ and ^3^A″ triplet states that correlate with the O_2_(*a*^1^Δ_*g*_) and O(^3^*P*) fragments approximates the overall probability of transitions to ^3^A′ versus ^3^A″ states, which corresponds to the relative probability of forming even and odd rotational states, respectively. As discussed previously, the O_2_ states with A′ symmetry must correspond to even rotational states and the states with A″ symmetry must correspond to odd. The O_2_(*a*^1^Δ_*g*_) fragment was previously shown to conserve the symmetry of the parent O_3_ following Hartley band dissociation at cold temperatures,^11,33^ so the ratio of even and odd rotational states of O_2_(*a*^1^Δ_*g*_) should reflect the crossing probability of the parent O_3_ to ^3^A′ and ^3^A″ states. Using the Landau–Zener model for a 320 nm dissociation and couplings from Grebenshchikov and Rosenwaks, the overall probability of transitions from the B state to ^3^A″ state is predicted to be 1.55 times higher than transitions from the B state to ^3^A′ state for the geometry used in the calculations, which would result in increased populations of odd rotational states. Additional details about the calculations are included in the Supporting Information. The intensity of the even and odd transitions in the 320 nm REMPI spectrum indicate that both A′ and A″ states contribute to the formation of O_2_(*a*^1^Δ_*g*_) products, and the A″ states dominate. Despite the simplicity of this analysis, it provides a very reasonable agreement with the observed enhancement of the odd peaks in the 320 nm spectrum in Figure 2 relative to the 266 nm spectrum.

Ion images can be used to further study dissociation dynamics through fitting the angular distributions to obtain vector correlations between the parent transition dipole moment **μ**, the fragment velocity ***v***, and the fragment angular momentum ***j***, which provide information about the dynamics of the dissociation. In the Hartley band, differences in the vector correlations between the even and odd rotational states of O_2_(*a*^1^Δ_*g*_) support the Λ-doublet model.^11^ In a typical two-color experiment, images are collected in three geometries (VV, HV, and VH, where V and H indicate polarization of the photolysis and probe lasers parallel and perpendicular to the imaging plane, respectively), and the angular distributions of each image are fit to obtain bipolar moments using the equations of Wei et al.^51^ Following the semiclassical bipolar moment formalism of Dixon, the low-order bipolar moments β_0_^2^(20), β_0_^0^(22), and β_0_^2^(02) represent the expectation value of the second Legendre polynomial ⟨*P*_2_(*cos θ*)⟩ where θ is the angle between the **μ** – ***v***, ***v*** – ***j***, and **μ** – ***j*** vectors, respectively.^52^ According to this formalism, the bipolar moment will be −0.5 if the vectors are perpendicular, and 1 if the vectors are parallel. The well-known spatial anisotropy parameter β is related to β_0_^2^(20) by the relation β = 2β_0_^2^(20). Image angular distributions are reported in terms of the image anisotropy parameters β_2_ and β_4_ rather than β_0_^2^(20), β_0_^0^(22), and β_0_^2^(02) because of the limitations of the vector correlation analysis possible in a one-color experiment. The reported values of β_2_ and β_4_ are related to the bipolar moments through the equations of Wei et al.^51^

**Figure 3:**
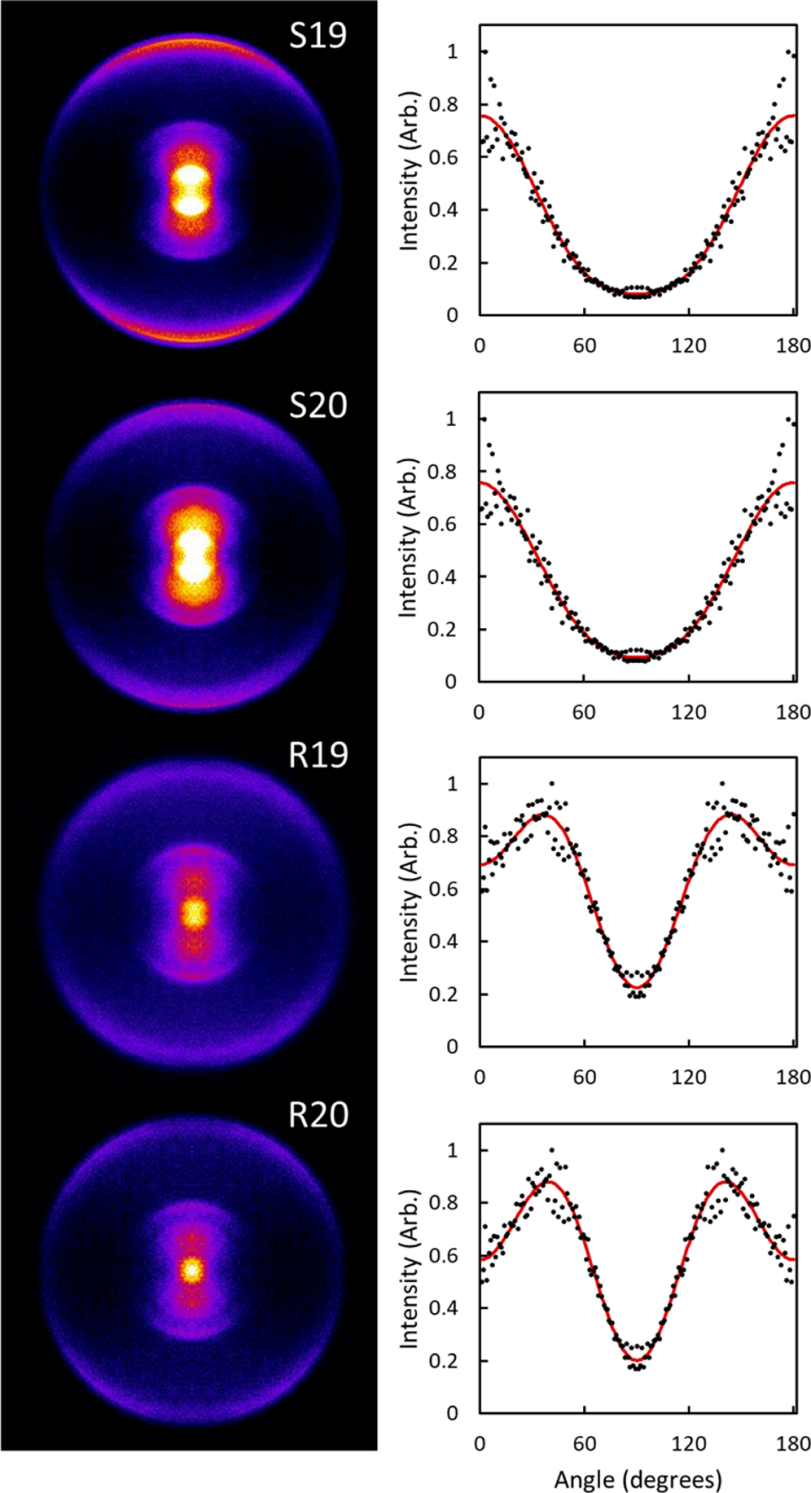
Symmetrized images of O_2_(*a*^1^Δ_*g*_, v = 0) following dissociation of O_3_ near 320 nm. A single, vertically polarized laser was used for both dissociation and probing the O_2_ fragments at wavelengths corresponding to the S- and R-branch transitions of *j* = 19 and *j* = 20. The outer ring corresponds to spin-forbidden dissociation, and the innermost ring is energetically consistent with spin-allowed dissociation from vibrationally excited O_3_. The signal at intermediate radii is nonresonant. Angular distributions correspond to the outermost ring in the images. Black circles are the experimental angular distributions, and red lines are Legendre polynomials (eq 7) with the best-fit anisotropy parameters β_2_ and β_4_ reported in Table 1.

Figure 3 shows ion images of O_2_(*a*^1^Δ_*g*_) in *j* = 19 and 20 following dissociation of O_3_ near 320 nm. These images were collected in a single laser experiment with vertical polarization at wavelengths corresponding to the R- and S-branch transitions of *j* = 19 and 20. As discussed above, the outermost ring in the images corresponds to the highest fragment speed, associated with the spin-forbidden dissociation channel (4). The signal in the image at radii less than half the outer ring is associated with spin-allowed dissociation of vibrationally excited O_3_ based on its translational energy. The signal at intermediate radii corresponding to approximately half the speed of the outer ring is diffuse, nonresonant, and is largely independent of wavelength. Images were symmetrized and reconstructed, and a narrow radial range of the outer ring was integrated to obtain the angular distributions shown on the right in Figure 3. The experimental angular distributions are shown in black circles, and the red line is fit to the equation
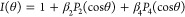
7to obtain the image anisotropy parameters, β_2_ and β_4_, where *I*(θ) is the angle-dependent intensity and *P*_2_ and *P*_4_ are the second and fourth Legendre polynomials, respectively. The values of β_2_ and β_4_ are reported in Table 1.

**Table 1:** Image Anisotropy Parameters for the Angular Distributions in Figure 3 Obtained by Fitting Image Angular Distributions Shown in Figure 3 with the Equation *I*(θ) = 1 + β_2_*P*_2_(cos θ) + β_4_*P*_4_(cos θ)

state	β_2_	β_4_
S19	1.67 ± 0.06	0.44 ± 0.10
S20	1.54 ± 0.07	0.33 ± 0.13
R19	0.78 ± 0.06	–0.61 ± 0.08
R20	0.75 ± 0.05	–0.74 ± 0.06

The intensity at the top and bottom of the images and the positive β_2_ values are indicative of a parallel transition, as expected for transition to the B state of O_3_. Because excitation in both the Hartley and Huggins bands is a parallel transition to the B state, the **μ**-***v*** correlation should be positive. The outer rings in the S-branch images show 2-fold symmetry, and the R-branch images show 4-fold symmetry, consistent with a perpendicular ***v***-***j*** correlation and expected for a triatomic dissociation. Following 266 nm dissociation, the anisotropy parameters and corresponding vector correlations are similar for odd and even rotational states at low temperatures but differ significantly at warmer temperatures.^11^ The values of β_0_^0^(22) and β_0_^2^(02) for *j* = 19 are closer to zero at higher temperatures due to greater depolarization of the ***v***-***j*** and **μ**-***j*** correlations because the odd rotational states of O_2_ originate from warmer parent molecules with greater angular momentum. Increased out-of-plane rotation decreases the angle between ***v*** and ***j***. Although the differences are more significant at higher temperatures, differences between β_0_^0^(22) and β_0_^2^(02) for even and odd rotational states can be seen across a range of temperatures.

Complete analysis of the 320 nm image anisotropy parameters to extract vector correlations is not possible because the images were limited to a single geometry, but the similarity between the angular distributions and image anisotropy parameters of odd and even rotational states is indicative of a cold molecular beam, consistent with the NO calibration previously discussed. This supports the explanation that the increased intensity of the odd rotational peaks in Figure 2 is a result of differences in ^3^A′ and ^3^A″ coupling to the B state rather than a depolarization mechanism from a warmer distribution of O_3_. Furthermore, given the hypothesis that the even and odd states originate from dissociation along the ^3^A′ and ^3^A″ repulsive states, respectively, the results imply similar dynamics on these surfaces, as expected.

#### 3.1.2 O_2_(*a*^1^Δ_*g*_, v = 0) Rotational Distribution at 320 nm

**Figure 4:**
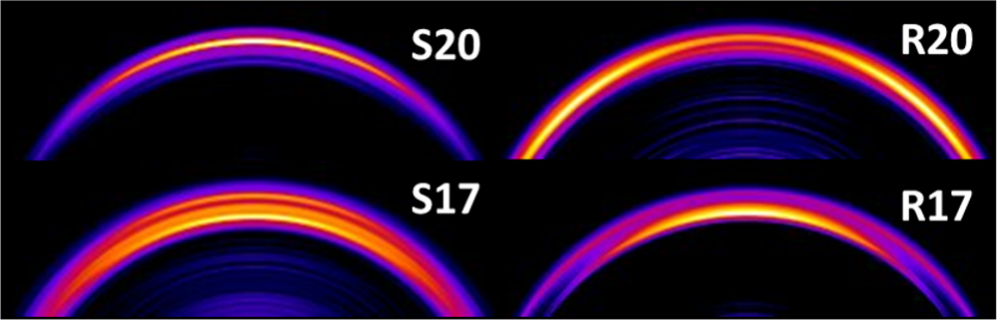
Outer edge of the reconstructed images of O_2_(*a*^1^Δ_*g*_) following 320 nm dissociation of O_3_ are shown. Images taken at wavelengths corresponding to an S-branch transition of *j* = 20 (top left) and R-branch transition of *j* = 20 (top right) have a single outer ring. Images taken at wavelengths corresponding to the S-branch transition of *j* = 17 (bottom left) and the R-branch transition of *j* = 17 (bottom right) have multiple rings, indicative of overlapped rotational transitions.

The 2–0 band of the O_2_(*d*^1^Π_*g*_ ←← *a*^1^Δ_*g*_) transition was previously measured by Gunthardt et al. to study the temperature-dependent alternation between odd and even rotational states of O_2_ following a 266 nm dissociation since the perturbed 2–0 transition exhibited more intense peaks corresponding to odd rotational states than unperturbed vibrational bands.^11^ Although the perturbations limit the use of the 2–0 REMPI spectrum to obtain accurate rotational state populations, we find strong evidence to suggest that the O_2_(*a*^1^Δ_*g*_) rotational distribution at 320 nm is broader than that at 266 nm. Han et al. employed the unperturbed 4–0 band instead to obtain the rotational state distribution of O_2_(*a*^1^Δ_*g*_) following O_3_ dissociation at 266 nm and found population in rotational states ranging from *j* = 13 to *j* = 37.^33^ The perturbations in the 2–0 band lead to several overlapping peaks in the REMPI spectrum that correspond to a combination of a low *j* and high *j* states from different branches which have been assigned by Morrill et al.^53^ Although the R and S branches for *j* = 15–21 in the 2–0 spectrum are overlapped by transitions from *j* = 35 to *j* = 44, these states are not observed following 266 nm dissociation, consistent with their low populations and confirmed by the unimodality of the speed distributions.^11^ At 320 nm, however, the speed distributions in this region are multimodal, indicating contributions from fragments at multiple speeds. Figure 4 shows the outer edge of reconstructed images of O_2_(*a*^1^Δ_*g*_) following a 320 nm dissociation of O_3_ at wavelengths corresponding to S- and R-branch transitions of *j* = 17 and *j* = 20. While there is a single outer ring in the *j* = 20 images, the ring is split in the *j* = 17 images, indicative of overlapping transitions from different O_2_(*a*^1^Δ_*g*_) rotational states.

**Figure 5:**
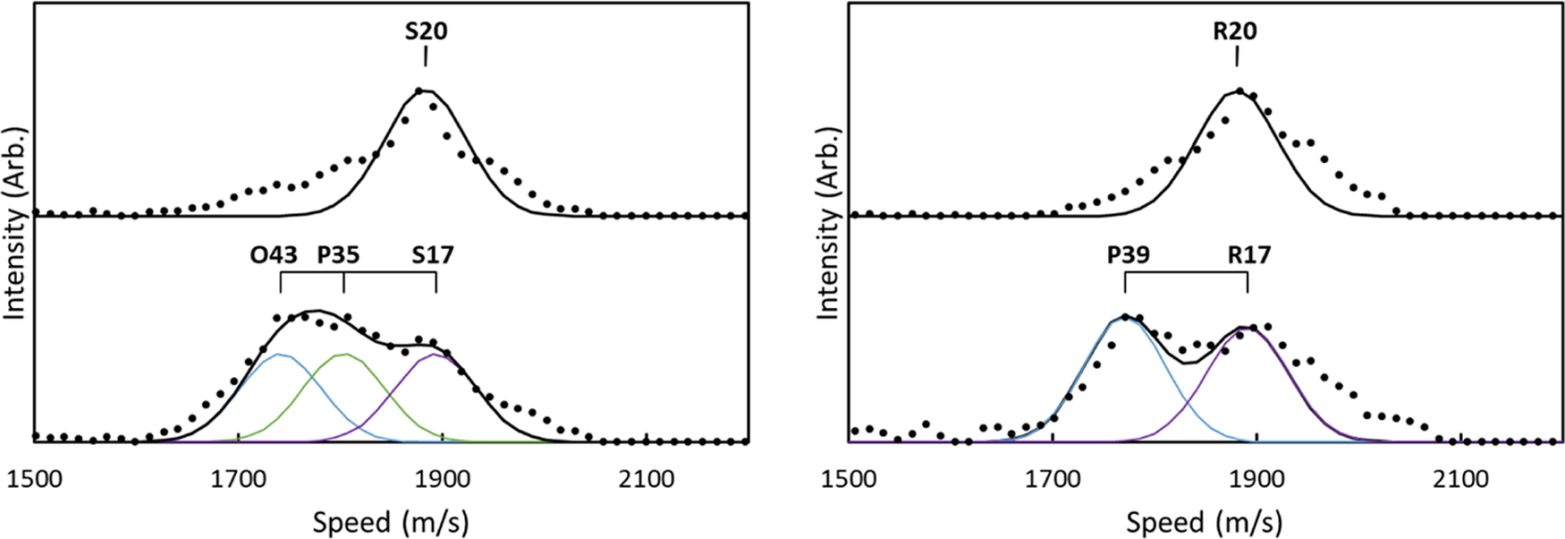
Speed distributions of O_2_(*a*^1^Δ_*g*_) extracted from the images shown in Figure 4. Distributions are shown for S- (left) and R-branch (right) images of *j* = 20 (top) and *j* = 17 (bottom). Images of both the S- and R-branch transitions of *j* = 20 are unimodal, indicating a single rotational state is observed at this wavelength. The image of the S-branch transition of *j* = 17 includes slow fragments attributed to the P-branch transition of *j* = 35 and the O-branch transition of *j* = 43, and the R-branch transition of *j* = 17 includes slow fragments assigned to the P-branch transition of *j* = 39.

Figure 5 shows the speed distributions derived from the reconstructed images in Figure 4. The speed distributions for *j* = 20 in both the S and R branches are unimodal, consistent with the distributions observed at 266 nm by Gunthardt et al.^11^ and indicative of a single rotational transition. In contrast, the speed distributions for *j* = 17 in both the S and R branches show additional contributions from slower fragments, indicating the presence of higher rotational states. The difference in energy of the fragments is consistent with the *j* = 35 (P-branch) and *j* = 43 (O-branch) transitions overlapping with the *j* = 17 (S-branch) transition and the *j* = 39 (P-branch) transition overlapping with the *j* = 17 (R-branch) transition, which are predicted based on the assignments of Morrill et al.^53^ The primary source of broadening in the speed distributions is instrumental uncertainty, and the speed distributions have been fit with Gaussian distributions with σ = 40 m/s, centered at the expected speeds based on the fragment rotational states. Additional images and speed distributions for *j* = 18 and 19 are included in the Supporting Information and also show evidence for *j* > 30 rotational states.

The fragment rotational distribution is a function of both the available energy and the dynamics of the dissociation, and while quantitative rotational state populations cannot be obtained from the O_2_(*d*^1^Π_*g*_, v = 2 ←← *a*^1^Δ_*g*_, v = 0) REMPI spectrum and images, it is clear that higher rotational states are populated following a 320 nm spin-forbidden dissociation than observed following a 266 nm spin-allowed dissociation. Additional direct evidence for the population of high rotational states is seen in the S-branch REMPI spectrum in Figure 2. In the REMPI spectrum following 266 nm dissociation at a warmer temperature (210 K), *j* = 21 is a minor shoulder on the *j* = 20 peak,^11^ whereas there is a pronounced peak in the REMPI spectrum at slightly shorter wavelengths than the *j* = 20 peak following 320 nm dissociation. This suggests the peak is primarily from the *j* = 42 P-branch transition which is not populated following 266 nm dissociation rather than the *j* = 21 S-branch transition.

The angular distributions of the rings corresponding to the R-branch transitions of *j* = 17 and *j* = 20 have 4-fold symmetry and the S-branch transitions have 2-fold as expected for a dissociation with ***v*** perpendicular to ***j***. The slower fragments in the S-branch image of *j* = 17 have 2-fold symmetry. This is consistent with the assignment of the O-branch transition of *j* = 43, but the P-branch transition of *j* = 35 is expected to have 4-fold symmetry. It is likely the 2-fold symmetry from the O-branch and S-branch transitions are dominant. In the image of the R-branch transition of *j* = 17, both the R-branch transition of *j* = 17 and the P-branch transition of *j* = 39 are expected to have 4-fold symmetry as seen in the R-branch images in Figure 3, but the angular distribution of the slower fragments has 2-fold symmetry. This suggests the presence of an overlapping, yet unassigned, O- or S-branch transition at this wavelength. Based on the assignments of nearby peaks by Morrill et al.,^53^ it is possible the O-branch transitions of *j* = 40–42 are in this energy range and would contribute to the observed 2-fold symmetry while still accounting for the observed velocity distribution because of the similarity in velocity for fragments in *j* = 39 and *j* = 40 states.

### 3.2 Photodissociation near 330 nm

#### 3.2.1 O_2_(*a*^1^Δ_*g*_) Rotational Distribution at 330 nm

**Figure 6:**
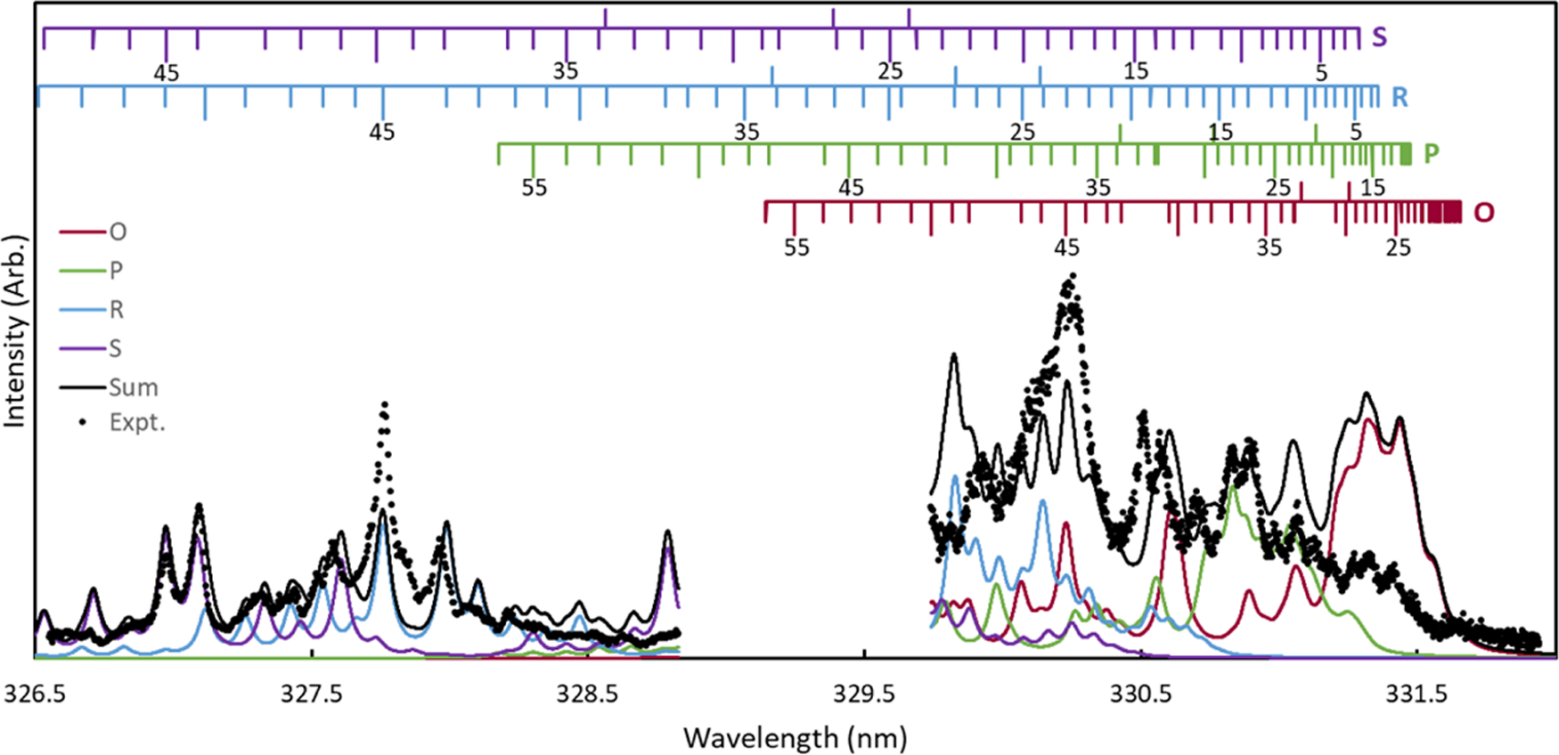
REMPI spectrum of O_2_(*a*^1^Δ_*g*_) following photodissociation of O_3_ in one-color experiments near 330 nm, probed via the 1–0 band of the *d*^1^Π_*g*_ ←← *a*^1^Δ_*g*_ transition. Black circles indicate experimental data. The data at shorter wavelengths were collected in the 2D-REMPI in Figure 7, and a narrow range of speeds was integrated to obtain the O_2_(*a*^1^Δ_*g*_, v = 0) spectrum. The longer-wavelength spectrum was collected in a one-color, 1D-REMPI experiment. The maroon, green, blue, and purple lines indicate the O, P, R, and S branches, respectively, and the solid black line is the sum of the branches.

**Figure 7:**
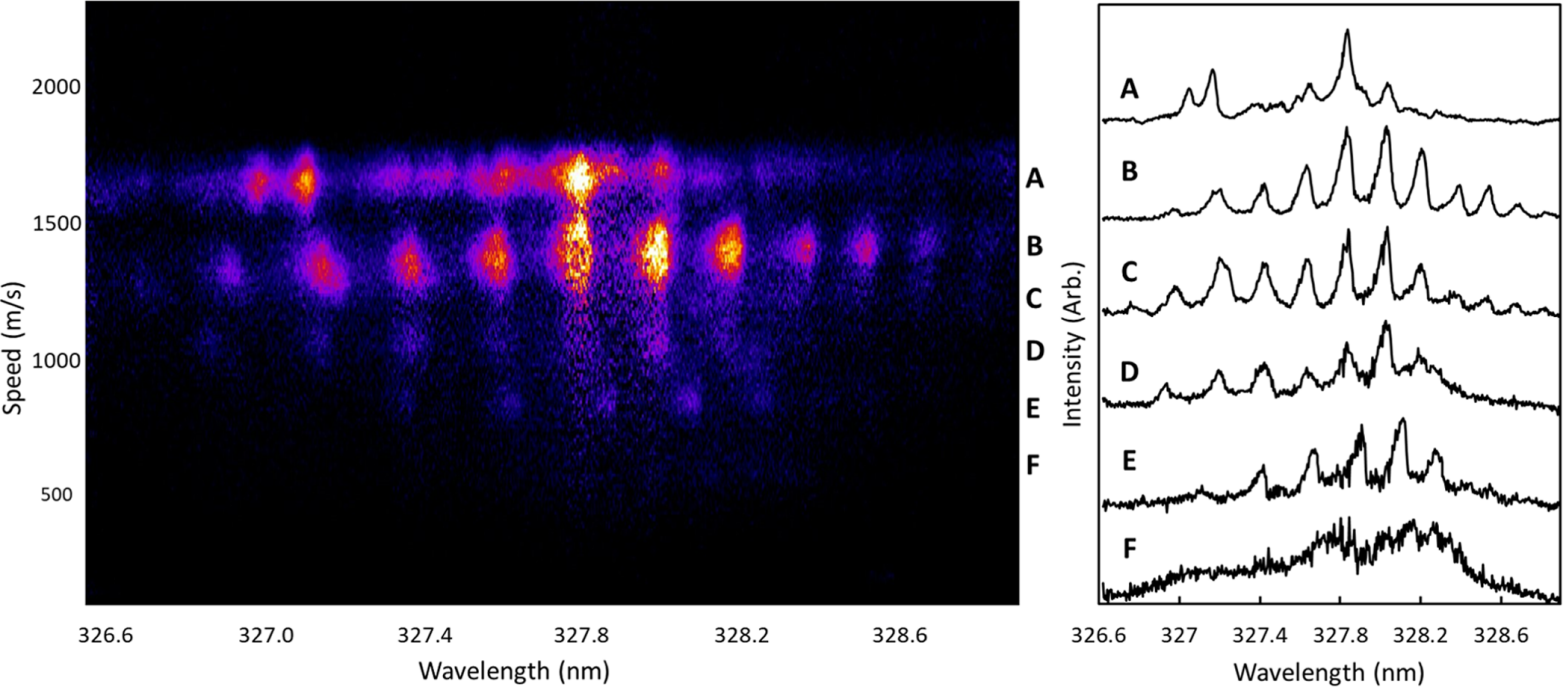
2D-REMPI spectrum of O_2_(*b*^1^Σ_*g*_^+^) and O_2_(*a*^1^Δ_*g*_) following the 330 nm dissociation of jet-cooled O_3_. The regions indicated by A–F were radially integrated to obtain the 1D spectra shown on the right. Region A is assigned to O_2_(*a*^1^Δ_*g*_, v = 0), and regions B–F are assigned to (*b*^1^Σ_*g*_^+^, v = 0–4) from top to bottom.

Figure 6 shows the REMPI spectrum of O_2_(*a*^1^Δ_*g*_) probed via the 1–0 band of the O_2_(*d*^1^Π_*g*_ ←← *a*^1^Δ_*g*_) transition in a one-color experiment near 330 nm. This spectrum supports a broad rotational distribution, consistent with conclusions based on the image speed distributions for a 320 nm dissociation. The experimental spectrum is shown in black circles. The spectrum at shorter wavelengths is from a 2D-REMPI spectrum (vide infra), integrated over a narrow range of speeds corresponding to the O_2_(*a*^1^Δ_*g*_, v = 0) fragment (region A in Figure 7). The spectrum at longer wavelengths is a traditional 1D-REMPI spectrum in which the center of the detector was covered with a mask, and the total signal was collected with a PMT, without radial resolution. The O, P, R, and S branches are shown in maroon, green, blue, and purple, respectively, with the sum of the branches shown by the solid black line. The transition energies used in the fit were determined from the rotational levels of the O_2_(*d*^1^Π_*g*_, v = 1) state reported by O’Keeffe et al.^13^ and fitting the rotational levels of O_2_(*a*^1^Δ_*g*_, v = 0) reported by Morrill et al.^53^ to obtain spectroscopic constants, which were adjusted to better fit a previous REMPI spectrum of O_2_(*a*^1^Δ_*g*_, v = 0) following 266 nm dissociation.^33^ No data were collected at intermediate wavelengths. Given that the two spectra were collected independently, they have been scaled to best fit the simulation. The spectrum is congested and highly perturbed, and the intensity of specific transitions cannot be directly used to extract rotational populations. We have attempted to correct transition intensities by including scaling factors for each rotational level of the resonant *d*^1^Π_*g*_ state that account for the perturbations by scaling all transitions that lead to the same final state equivalently based on fits to a known distribution (see the Supporting Information). Additionally, the high-intensity peaks at short wavelengths are at wavelengths similar to peaks in the Huggins band absorption spectrum of O_3_, which may indicate that the increase in intensity is due to increased O_3_ absorption rather than higher population in rotational states corresponding to the transitions at the high-intensity wavelengths. The increase in the signal may also indicate that the rotational distribution of O_2_(*a*^1^Δ_*g*_) and the branching ratio between the O_2_(*a*^1^Δ_*g*_) and the O_2_(*b*^1^Σ_*g*_^+^) may depend on whether the O_3_ dissociation is on- or off-resonance with a Huggins band peak. Determining accurate populations is challenging despite our efforts, but the spectrum clearly implies a broad rotational distribution with a significant population in states ranging from *j* ∼ 15 to *j* ∼ 50, consistent with the analysis at 320 nm. In particular, signal intensity between 331 and 331.5 nm corresponds to transitions for *j* ≤ 25, whereas signal intensity between 326.5 and 328 nm corresponds to transitions for *j* ≥ 40. Speed distributions of images collected at wavelengths within the range of this spectrum are also consistent with the assigned rotational states.

#### 3.2.2 O_2_(*b*^1^Σ_*g*_^+^) Rotational Distribution at 330 nm

In addition to studies on the spin-forbidden dissociation via channel 4 producing O_2_(*a*^1^Δ_*g*_) and O(^3^*P*) products, there have been previous studies on the O_2_(*b*^1^Σ_*g*_^+^) fragment formed in channel 3.^13,34^ The b^1^Σ_*g*_^+^ state of O_2_ is 5200 cm^–1^ higher in energy than the a^1^Δ_*g*_ state^53^ but with similar rotational constants.^34^ Because of symmetry restrictions, only even rotational states exist in the O_2_(*b*^1^Σ_*g*_^+^) state. Previous studies on the O_2_(*b*^1^Σ_*g*_^+^) fragment utilized REMPI schemes accessing the v = 1 or 2 state of the resonant *d*^1^Π_*g*_ state, which are highly perturbed, making the rotational state distributions difficult to obtain. Fortuitously, 330 nm corresponds to the 4–0 band of the O_2_(*d*^1^Π_*g*_ ←← *b*^1^Σ_*g*_^+^) transition, which accesses the lowest unperturbed vibrational level of the *d*^1^Π_*g*_ state, allowing the determination of accurate rotational state populations of the *b*^1^Σ_*g*_^+^ state, which can be compared to the *a*^1^Δ_*g*_ state populations.

Figure 7 shows a 2D-REMPI spectrum of O_2_ in a one-color experiment following dissociation of O_3_ near 330 nm. At each wavelength step in the scan, an image was collected, symmetrized, and reconstructed, and the reconstructed speed distribution was plotted as a function of wavelength to obtain the 2D spectrum. There are several structured bands clearly visible in Figure 7 at different speeds, which can be assigned to different electronic and vibrational states of O_2_ based on the speed of the fragments and energy conservation. The fragments with the greatest speed (top of the figure) correspond to v = 0 of the O_2_(*a*^1^Δ_*g*_) electronic state. The remaining signal is assigned to vibrational levels of the O_2_(*b*^1^Σ_*g*_^+^) state, with the higher vibrational states appearing at slower speeds due to the increased internal energy. Narrow ranges of speed were integrated to obtain the 1D spectra shown on the right, each corresponding to a different electronic or vibrational state of O_2_. Region A is the spectrum included in Figure 6 and discussed previously.

**Figure 8:**
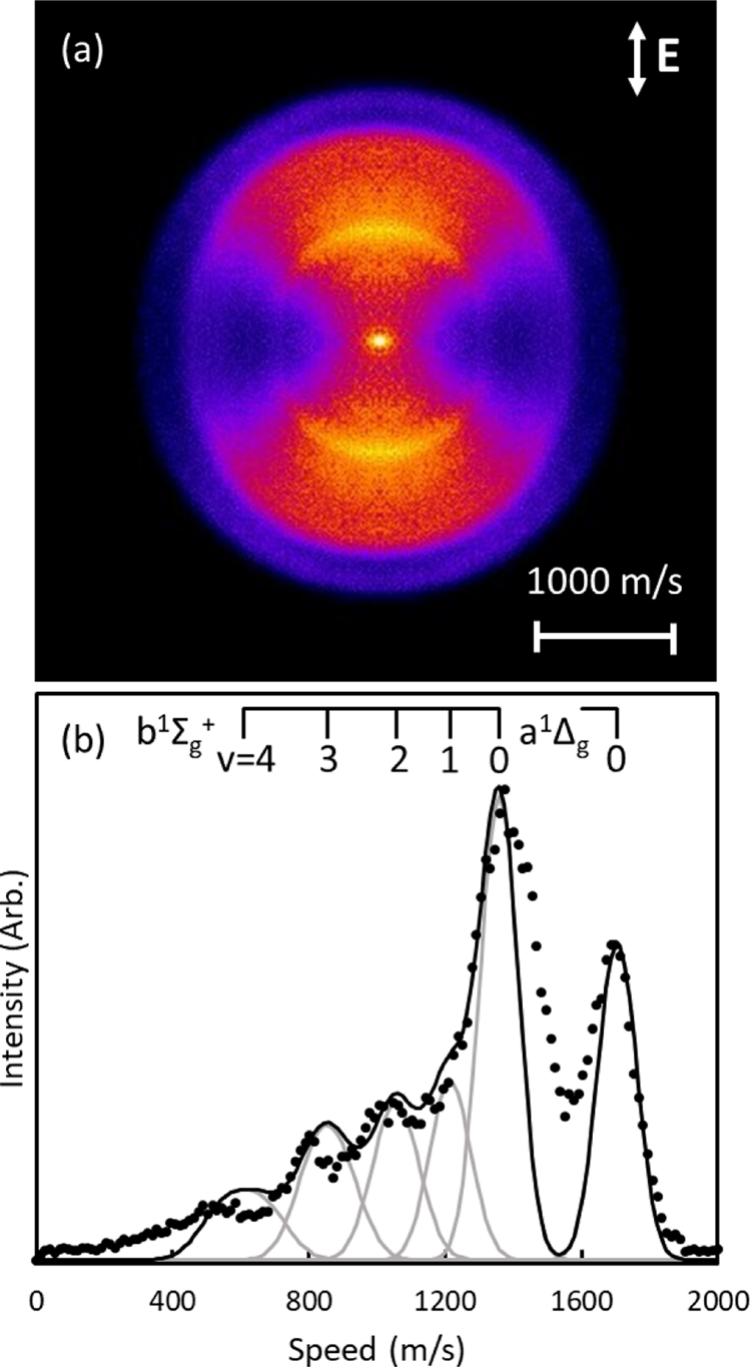
Image obtained from averaging all images collected in the 2D-REMPI spectrum in Figure 7 between 327.970 and 328.145 nm is shown above. This region was selected to include a single rotational peak from each vibrational level. The radial distribution of the averaged, reconstructed image is shown below. The speed distribution was fit with a sum of Gaussian distributions, with peaks corresponding to the speeds expected for the vibrational and rotational states included in the narrow-wavelength region.

Figure 8 shows an averaged image and corresponding speed distribution of a narrow slice of the 2D-REMPI, integrated between 327.970 and 328.145 nm. The speed distribution shows clear structure corresponding to O_2_(*a*^1^Δ_*g*_, v = 0) and O_2_(*b*^1^Σ_*g*_^+^, v = 0–4) products. The distribution has been fit with Gaussian distributions centered on the speeds expected for each vibrational and rotational state detected in the integrated wavelength region. The peak at 1700 m/s corresponds to v = 0 of O_2_(*a*^1^Δ_*g*_). The peak at 1350 m/s corresponds to O_2_(*b*^1^Σ_*g*_^+^, v = 0), and O_2_(*b*^1^Σ_*g*_^+^, v = 1) appears as a shoulder on the v = 0 peak, indicating there is overlap between these two vibrational levels in the 1D rotational spectra shown in Figure 7. The other peaks in the speed distribution are consistent with the speeds expected for fragments in v = 2, 3, and 4 of O_2_(*b*^1^Σ_*g*_^+^). The Gaussian distributions used to fit the speed distribution have increasing values of σ for the higher vibrational states to reflect the effect of similar energy distributions having broader speed distributions at low speeds.

**Figure 9:**
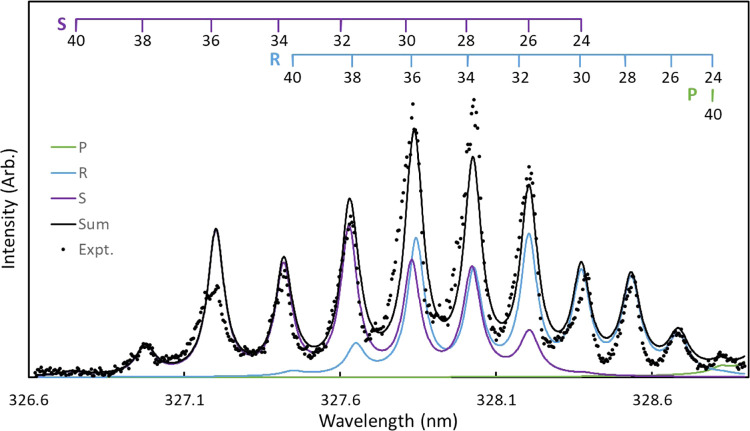
1D rotational spectrum of O_2_(*b*^1^Σ_*g*_^+^, v = 0) obtained by integrating a narrow range of speeds in the 2D-REMPI spectrum corresponding to region B in Figure 7. These wavelengths correspond to the 4–0 band of the O_2_(*d*^1^Π_*g*_ ←← *b*^1^Σ_*g*_^+^) transition. The experimental data are shown by black circles, and the P, R, and S branches of the simulated spectrum are shown in green, blue, and purple, respectively. The solid black line represents the sum of all of the simulated branches.

**Figure 10:**
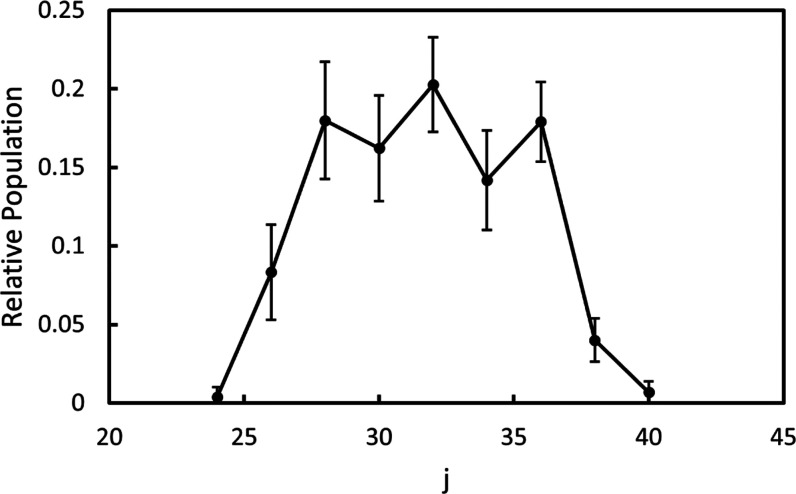
Rotational state distribution of O_2_(*b*^1^Σ_*g*_^+^, v = 0) fit to the REMPI spectrum shown in Figure 9. Only even rotational states of O_2_(*b*^1^Σ_*g*_^+^) are permitted due to symmetry restrictions.

The 1D rotational spectrum generated by integrating the 2D-REMPI over a narrow range of speeds can be fit to obtain rotational state populations for individual vibrational states. Figure 9 shows the 1D spectra of O_2_(*b*^1^Σ_*g*_^+^, v = 0). The REMPI spectrum was fit with a simulation using spectroscopic constants fit to transitions previously reported by O’Keeffe et al. for the b^1^Σ_*g*_^+^ state^13^ and constants from Morrill et al. for the d^1^Π_*g*_ state.^53^ The simulation also includes two-photon line strengths from Bray and Hochstrasser.^54^ Linewidths in Figure 9 are based on previously reported *j*-dependent line width trends by Aardema et al.,^31^ slightly shifted to larger linewidths because of broadening as a result of additional laser power. The simulated P, R, and S branches in Figure 9 are shown in green, blue, and purple, respectively, and the sum of the branches is shown by the solid black line. The experimental data is represented by black circles. The rotational state distribution used in the fit is shown in Figure 10, and the error bars were obtained by a Monte Carlo simulation. The rotational state distribution is slightly narrower but overall consistent with the distributions reported by O’Keeffe et al. following O_3_ dissociation at 337.2 and 344 nm.^13^ In comparison to the range of rotational states of O_2_(*a*^1^Δ_*g*_, v = 0) observed following both 320 and 330 nm dissociation of O_3_, the rotational distribution of the O_2_(*b*^1^Σ_*g*_^+^, *v* = 0) is much narrower, indicative of significantly different dynamics in the dissociation processes leading to each of the electronic channels. While there is clear evidence that the rotational distribution of O_2_(*a*^1^Δ_*g*_) extends from at least *j* = 16 to j = 43 following dissociation at both 320 and 330 nm, there is only population in *j* = 24–40 of the *b*^1^Σ_*g*_^+^ state. A narrow rotational distribution could be an indication of anisotropy in the potential or selectivity of molecules with a limited range of geometries that can transition from the B state of O_3_ to the triplet states correlating with the O_2_(*b*^1^Σ_*g*_^+^) and the O(^3^*P*) products, while molecules with a wider range of bond angles can transition from the B state to triplet states correlating with O_2_(*a*^1^Δ_*g*_) and O(^3^*P*) products, leading to a broad rotational state distribution of O_2_(*a*^1^Δ_*g*_). In the analysis of O(^3^*P*) images following spin-forbidden dissociation of O_3_ by Ulrich et al., the authors assumed a single rotational temperature for each of the three O_2_ electronic states that were produced with an O(^3^*P*) co-fragment.^6^ The difference in the width of the rotational distributions measured for O_2_(*a*^1^Δ_*g*_) and O_2_(*b*^1^Σ_*g*_^+^) indicates that the two distributions cannot be described by a single temperature.

The slower fragments in the 2D-REMPI are assigned to the 5–1, 6–2, 7–3, and 8–4 bands of the O_2_(*d*^1^Π_*g*_ ←← *b*^1^Σ_*g*_^+^) transition. The spacing of the peaks is consistent with the presence of only even rotational states expected for the O_2_(*b*^1^Σ_*g*_) state, and the radial distribution is consistent with the expected speeds of the O_2_(*b*^1^Σ_*g*_^+^) fragments in higher vibrational states, as seen in Figure 8. The lower vibrational levels (v = 0–2) of the O_2_(*b*^1^Σ_*g*_^+^) state have been well characterized and rotational transitions have been assigned.^13,53^ Experimental rotational constants for v = 3 and v = 4 have not been reported to our knowledge but can be reasonably estimated from the lower vibrational levels. In the resonant O_2_(*d*^1^Π_*g*_) state, however, rotational analysis has not been performed for v ≥ 5, and estimating rotational constants from the lower vibrational levels is not possible because v = 0–3 are highly perturbed. Rotational constants were calculated using the BCONT program^55^ which determines the eigenvalues for an assumed potential which was adjusted to fit the experimental spectra. We found that the vibrational levels of the *d*^1^Π_*g*_ state needed to be shifted to lower energies than those predicted by the BCONT calculations and previously estimated by theory or from kinetic energy release spectra.^56^

**Figure 11:**
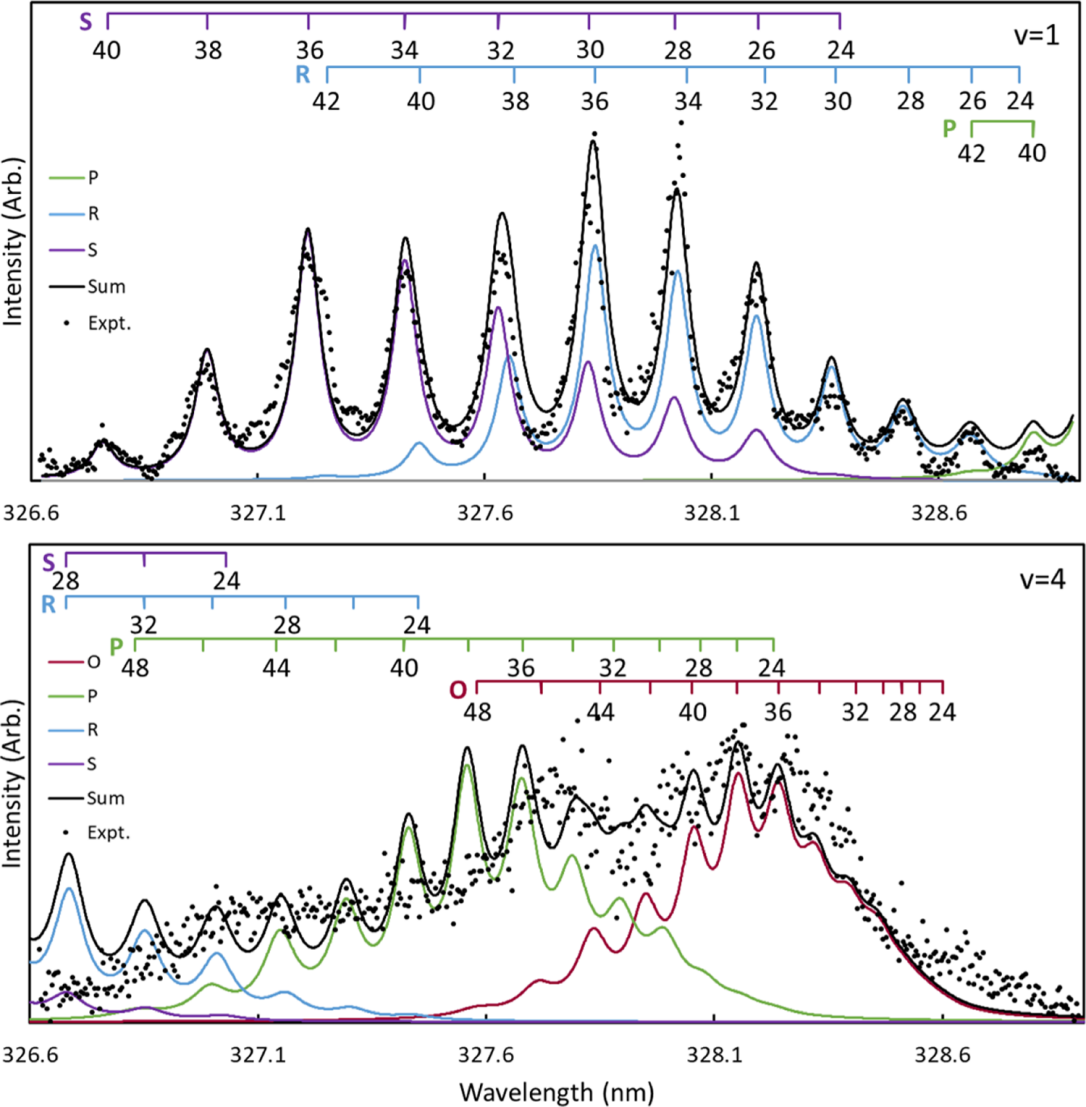
1D rotational spectra for the 5–1 (top) and 8–4 (bottom) bands of the O_2_(*d*^1^Π_*g*_ ←← *b*^1^Σ_*g*_^+^) transition following the dissociation of O_3_ near 330 nm. The rotational spectra correspond to regions C and F of Figure 7. The experimental spectra are represented by black circles, and the simulated O, P, R, and S branches are shown in maroon, green, blue, and purple, respectively. The sum of the branches is represented by the solid black line.

REMPI spectra for the 5–1 and 8–4 bands of the O_2_(*d*^1^Π_*g*_ ←← *b*^1^Σ_*g*_^+^) transition are shown in Figure 11 as representative examples of fits to the rotational spectra for higher vibrational states. Fits to the spectra for the 6–2 and 7–3 transitions are included in the Supporting Information. The black circles again represent the experimental data, and the simulated O, P, R, and S branches are shown in maroon, green, blue, and purple, respectively, with the sum of the branches shown by the solid black line. The signal at speeds corresponding to O_2_(*b*^1^Σ_*g*_^+^, v = 4) exhibits a very weak structure, which is a consequence of the low signal and overlapping branches, but there is clear evidence for the presence of v = 4 in the radial distribution and simulations to the spectrum provide a reasonable fit.

**Figure 12:**
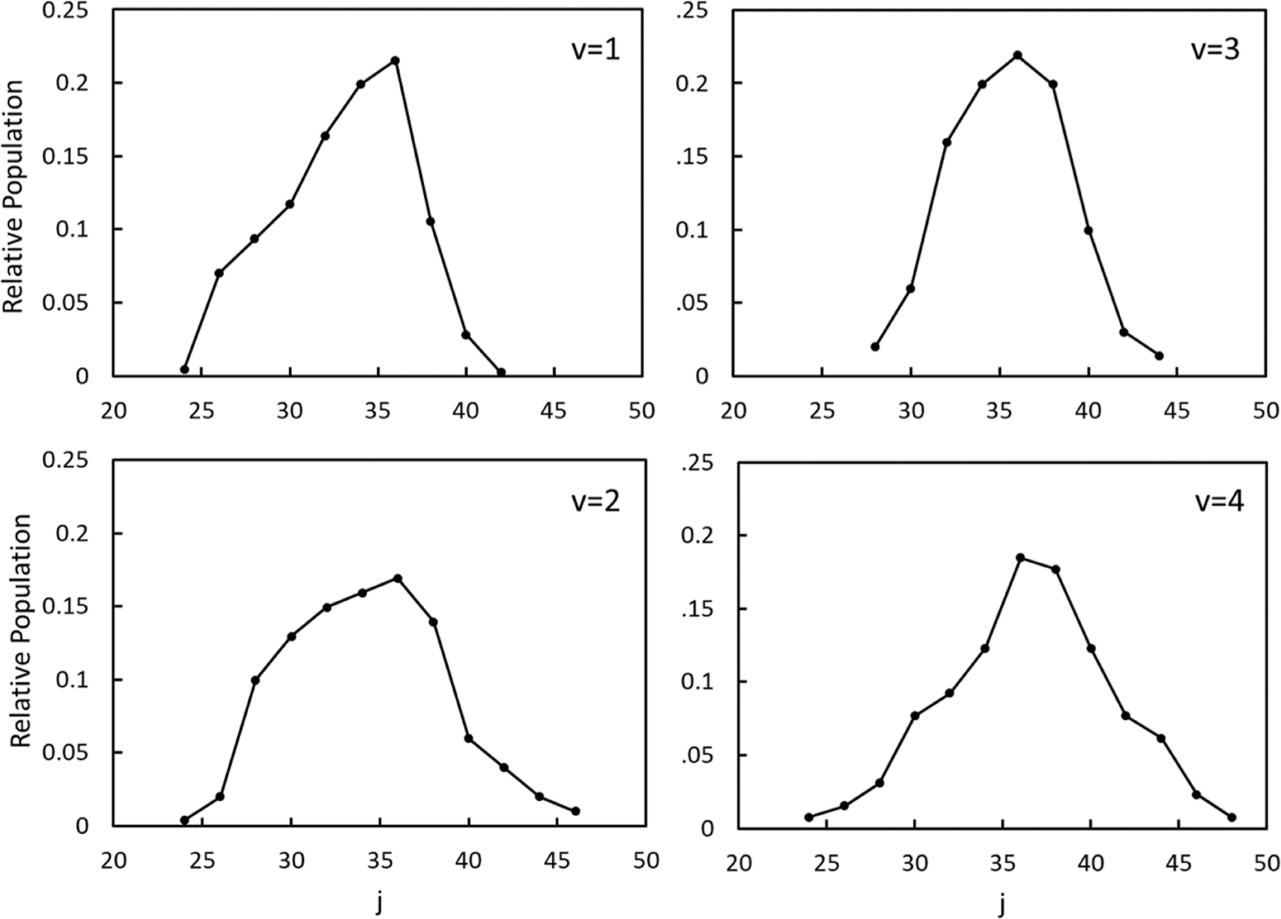
Rotational state populations for O_2_(*b*^1^Σ_*g*_^+^, v = 1–4) following the dissociation of O_3_ near 330 nm used to fit the rotational spectra in Figures 7 and 11.

The rotational distributions of O_2_(*b*^1^Σ_*g*_^+^, v = 1–4) used to fit the rotational spectra in regions C–F of Figure 7 are shown in Figure 12. The Supporting Information contains additional discussion of the fits to the spectra for v = 1–4 of the O_2_(*b*^1^Σ_*g*_^+^) fragment. It is clear that the rotational distributions for vibrational states 1–4 of O_2_(*b*^1^Σ_*g*_^+^) are very similar to that of v = 0, shifting to slightly higher rotational states with increasing vibrational state.

**Figure 13:**
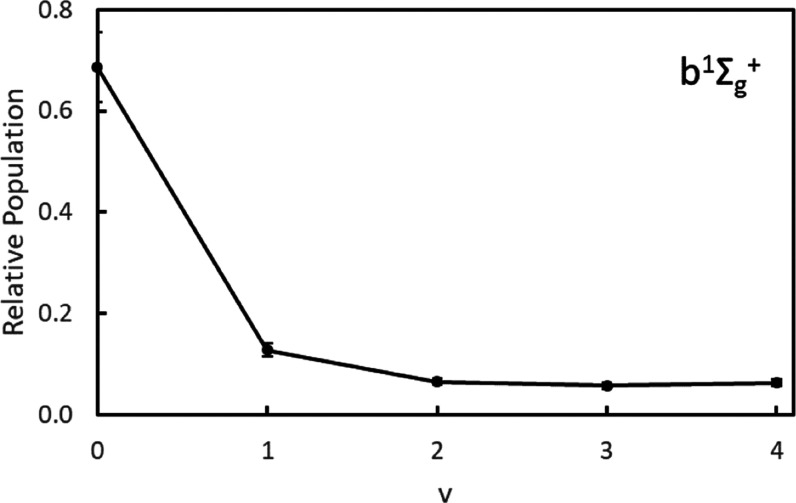
Vibrational distribution of the O_2_(*b*^1^Σ_*g*_^+^) fragment following 330 nm dissociation of O_3_, based on analysis of the speed distribution of the integrated 2D-REMPI spectrum and correcting for the Franck–Condon factor of each vibrational transition to the resonant *d*^1^Π_*g*_ state.

Because v = 0–4 of O_2_(*b*^1^Σ_*g*_^+^) are all probed simultaneously in the 2D-REMPI, the vibrational distribution can also be determined from the speed distribution of the spectrum. The 2D-REMPI was integrated from 327.620 to 328.870 nm to obtain the speed distribution of nearly the entire spectrum. The wavelength range was chosen to maximize R- and S-branch contributions and minimize P-branch contributions in O_2_(*b*^1^Σ_*g*_^+^, v = 0–3). The integrated speed distribution was fit with a Gaussian distribution for each vibrational level, centered at the speeds expected for a fragment in the rotational state in the middle of the rotational distribution for the given vibrational level. The Gaussian distributions were broader than the distributions in Figure 8 due to the increased range of speeds expected for a range of rotational states. The distributions were converted to energy, and the integrated areas of the fit Gaussian distributions were corrected for the Franck–Condon factors for each vibrational band of the O_2_(*d*^1^Π_*g*_ ←← *b*^1^Σ_*g*_^+^) transition to determine the population in each vibrational state. The resulting vibrational distribution is shown in Figure 13. The distribution peaks at v = 0 and decreases monotonically with increasing v, with very low populations at the higher vibrational states. Franck–Condon factors associated with the transitions increase with increasing initial vibrational level, which allows the detection of fragments in higher vibrational states despite their low population. The observed vibrational distribution is significantly different than the vibrational distribution reported by Ulrich et al. based on fits to the O(^3^*P*_2_) speed distributions, which predicts a maximum population in v = 2 for their “full” model and v = 1 for their “restricted” model for dissociation at 328.022 nm.^6^ Neither model predicts any population in v = 3 or 4, which is clearly observed in the 2D-REMPI spectrum, albeit with low populations. The measured vibrational state distribution for O_2_(*b*^1^Σ_*g*_^+^) is similar to the distribution calculated for the O_2_(X^3^Σ_*g*_^–^) fragment by Ulrich et al., which also peaks at v = 0 and decreases monotonically.^6^

Although there is no evidence in the 2D-REMPI for higher vibrational states of O_2_(*a*^1^Δ_*g*_), it is unclear whether this is due to low population or shifts in wavelength for the REMPI transitions of higher vibrational states.

### 3.3 O_2_(*b*^1^Σ_*g*_^+^) Ion Images

**Figure 14:**
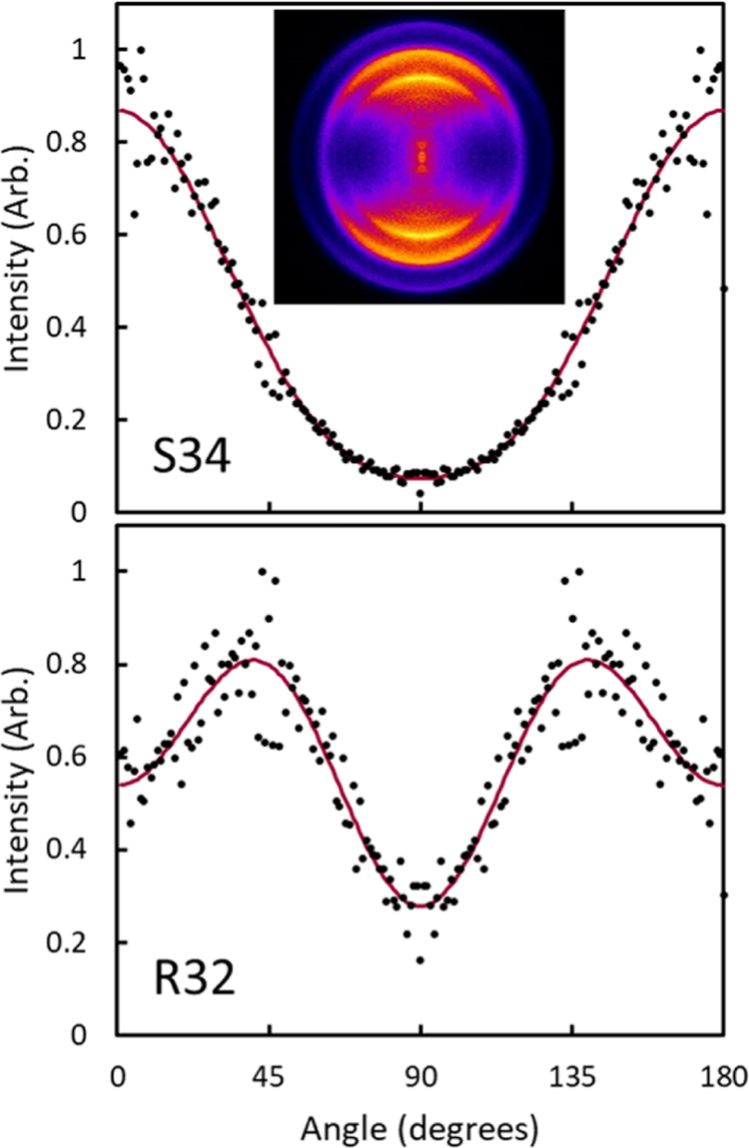
Angular distributions of ion images of O_2_(*b*^1^Σ_*g*_^+^, v = 0) collected in a one-color experiment following the dissociation of O_3_. The wavelengths correspond to the S-branch transition of *j* = 34 (top) and the R-branch transition of *j* = 32 (bottom). The image corresponding to the S-branch transition of *j* = 34 is included. Images were reconstructed, and a thin slice of the ring corresponding to the O_2_(*b*^1^Σ_*g*_^+^, v = 0) ring was fit to eq 7 to obtain β_2_ and β_4_ image anisotropy parameters in Table 2. Black circles represent the experimental angular distribution, and red lines represent the fit to eq 7. The faint outer ring in the image corresponds to the ionic ring of O_2_(*a*^1^Δ_*g*_, v = 0), and the rings at smaller radii correspond to higher vibrational states of O_2_(*b*^1^Σ_*g*_^+^).

Ion images of the O_2_(*b*^1^Σ_*g*_^+^) fragment were collected near 330 nm. As with experiments at 320 nm, quantitative extraction of vector correlations was limited due to the single laser geometry in a one-color experiment and images were only collected with the laser vertically polarized. Additionally, isolation of individual rotational states was difficult due to the considerable overlap between the R- and S-branch transitions. Figure 14 shows the angular distributions of images collected at wavelengths corresponding to the S-branch transition of *j* = 34 and the R-branch transition of *j* = 32 of O_2_(*b*^1^Σ_*g*_^+^, v = 0). Typically analysis is performed on both branches of a single rotational state, but due to the highly overlapped R and S branches, nearby rotational states were chosen that had minimal contributions from the other rotational branch. Based on the fit to the REMPI spectrum in Figure 9, the S-branch transition for *j* = 34 has very little contribution from the R-branch transition for *j* = 40. The *j* = 32 R-branch transition has some overlap with the *j* = 26 S-branch transition, but the R-branch, *j* = 32 transition should be dominant based on the fit to the spectrum in Figure 9. Images were collected with a single, vertically polarized laser and reconstructed, and a narrow slice of the ring corresponding to O_2_(*b*^1^Σ_*g*_^+^, v = 0) was used for the angular distribution. The faint outer ring in the image is from O_2_(*a*^1^Δ_*g*_, v = 0), and moving to smaller radii, the next ring is from O_2_(*b*^1^Σ_*g*_^+^, v = 0). Because the *b*^1^Σ_*g*_^+^, v = 0 ring in the image of the *j* = 32 R-branch transition contains contribution from the S-branch transition of *j* = 26, the edge of the ring at smaller radii was used for the angular distribution because fragments in *j* = 32 should have slightly slower speeds than fragments in *j* = 26. The outer edge of the ring was used for *j* = 34, which should be faster than *j* = 40. The angular distributions were fit to eq 7 to obtain the anisotropy parameters in Table 2. The angular distribution has 2-fold symmetry in the S-branch and 4-fold symmetry in the R-branch as expected for a perpendicular ***v***-***j*** correlation. The β_2_ parameter in the S-branch image is similar to the β_2_ parameters reported S-branch images of O_2_(*a*^1^Δ_*g*_), but β_2_ is lower in the R-branch image than O_2_(*a*^1^Δ_*g*_). The value of β_4_ is positive for the S-branch and negative for the R-branch, as seen in the ionization of the O_2_(*a*^1^Δ_*g*_) fragment.

**Table 2:** Image Anisotropy Parameters for the Angular Distributions in Figure 3 Obtained by Fitting Image Angular Distributions Shown in Figure 3 with the Equation *I*(θ) = 1 + β_2_*P*_2_(cos θ) + β_4_*P*_4_(cos θ)

state	β_2_	β_4_
S34	1.76 ± 0.08	0.40 ± 0.09
R32	0.56 ± 0.07	–0.62 ± 0.06

Using the R- and S-branch angular distributions to estimate bipolar moments gives β_0_^2^(20) near 0.5 and β_0_^0^(22) near −0.5, which is consistent with a parallel transition and perpendicular ***v***-***j*** correlation, as expected. The value of β_0_^2^(20) is expected to be diminished from its limiting value of 1 due to the lifetime of the excited state, which Takahashi et al. estimate to be between 0.3 and 1.1 ps for excitation near 325 nm.^23^ This is also consistent with the image anisotropy parameters reported by Ulrich et al. for rings in the O(^3^*P*_2_) images assigned to formation of an O_2_(*b*^1^Σ_*g*_^+^) co-fragment, which primarily range from 0.55 to 1.98, corresponding to β_0_^2^(20) values of 0.28 to 0.99.^6^ The image anisotropy parameters reported for the O_2_(*a*^1^Δ_*g*_) fragments similarly range from 0.61 to 1.99.

## 4 Conclusions

The REMPI spectrum of O_2_(*a*^1^Δ_*g*_) following 320 nm spin-forbidden dissociation of O_3_ probed via S-branch transitions of the O_2_(*d*^1^Π_*g*_, v = 2 ←← *a*^1^Δ_*g*_, v = 0) transition was reported, exhibiting greater intensity in peaks corresponding to odd rotational state transitions than even state transitions. While the observed alternation is opposite of that reported by Gunthardt et al. for the spin-allowed dissociation at 266 nm,^11^ it is consistent with the coupling between the B state and ^3^A′ and ^3^A″ states correlating to spin-forbidden products calculated by Grebenshchikov and Rosenwaks.^12^ We are optimistic that in the future it will be possible to study predissociation via the ^3^A′ and ^3^A″ states independently. Images of odd and even rotational states at 320 nm have very similar angular distributions, indicating similar dissociation dynamics. The radial distributions of the images collected at 320 nm are multimodal, and the speeds of the fragments are consistent with the overlap between low *j* and high *j* peaks assigned by Morrill et al.^53^ The radial distributions of these images indicate a broad rotational distribution of the O_2_(*a*^1^Δ_*g*_) fragment, which is also observed following dissociation near 330 nm in the 1D-REMPI spectrum. In contrast, the 2D-REMPI spectrum at 330 nm indicates a much narrower rotational distribution of O_2_(*b*^1^Σ_*g*_^+^) probed via the O_2_(*d*^1^Π_*g*_, v = 4 ←← *b*^1^Σ_*g*_^+^, v = 0) transition. There is evidence in the 2D-REMPI for the formation of higher vibrational states (v = 1–4) of O_2_(*b*^1^Σ_*g*_^+^) as well, with rotational distributions similar to v = 0. The radial distribution of the 2D-REMPI was used to obtain the vibrational state distribution of the O_2_(*b*^1^Σ_*g*_^+^) fragment, which is primarily formed in v = 0, with much less population in higher vibrational states. The difference in rotational distributions between the *a*^1^Δ_*g*_ and *b*^1^Σ_*g*_^+^ states of O_2_ indicates different dynamics leading to these dissociation channels, possibly with higher selectivity of the O_3_ bond angles in the channel forming O_2_(*b*^1^Σ_*g*_^+^) + O(^3^*P*), leading to a narrower rotational distribution.

Future two-color experiments in the Huggins band would allow not only full vector correlation analysis but also studies on the wavelength dependence of the O_2_(*a*^1^Δ_*g*_) and O_2_(*b*^1^Σ_*g*_^+^) rotational state distributions. Dissociation at wavelengths corresponding to the excitation of different vibrational modes may result in different fragment rotational state distributions or differences in the branching ratio between O_2_ electronic states, which are not distinguishable in the one-color experiments.

## Supporting Information Available

NO temperature calibration, a more detailed description of the Landau–Zener model used to estimate the relative probability of transitions from the B state to the 3A′ and 3A″ states, ion images and speed distributions for j = 17–20 in both the R and S branches, discussion of the scaling factors used in fitting the perturbed O_2_(*d*^1^Π*_g_*, v = 1 ←← a^1^Δ_*g*_, v = 0) REMPI spectrum, and the REMPI fits and rotational distributions for all O_2_(*b*^1^Σ*_g_*^+^) vibrational states obtained in the 2D-REMPI spectrum (PDF)

## References

[ref1] Johnston H. S. Atmospheric Ozone. Annu. Rev. Phys. Chem. 1992, 43, 1–32. 10.1146/annurev.pc.43.100192.000245. 18338974

[ref2] Grebenshchikov S. Y.; Qu Z.-W.; Zhu H.; Schinke R. New theoretical investigations of the photodissociation of ozone in the Hartley, Huggins, Chappuis, and Wulf bands. Phys. Chem. Chem. Phys. 2007, 9, 2044–2064. 10.1039/b701020f. 17464386

[ref3] Ball S. M.; Hancock G.; Pinot de Moira J. C.; Sadowski C. M.; Winterbottom F. Time-of-flight measurements of the kinetic energies of the *O*_2_(*a*^1^Δ_*g*_) fragment from the photolysis of ozone between 287 and 331 nm. Chem. Phys. Lett. 1995, 245, 1–6. 10.1016/0009-2614(95)01047-D.

[ref4] O’Keeffe P.; Ridley T.; Lawley K. P.; Maier R. R. J.; Donovan R. J. Kinetic energy analysis of *O*(^3^*P*) and *O*_2_(*b*^1^Σ_*g*_^+^) fragments produced by photolysis of ozone in the Huggins bands. J. Chem. Phys. 1999, 110, 10803–10809. 10.1063/1.479023.

[ref5] Takahashi K.; Kishigami M.; Matsumi Y.; Kawasaki M.; Orr-Ewing A. J. Observation of the spin-forbidden *O*(^1^*D*)+*O*_2_(*X*^3^Σ_*g*_^–^) channel in the 317–327 nm photolysis of ozone. J. Chem. Phys. 1996, 105, 5290–5293. 10.1063/1.472370.

[ref6] Ulrich C. K.; Chen J.; Tokel O.; Houston P. L.; Grebenshchikov S. Y. Photodissociation of Ozone from 321 to 329 nm: The Relative Yields of *O*(^3^*P*_2_) with *O*_2_(*X*^3^Σ_*g*_^–^), *O*_2_(*a*^1^Δ_*g*_) and *O*_2_(*b*^1^Σ_*g*_^+^). J. Phys. Chem. A 2013, 117, 12011–12019. 10.1021/jp4041088. 23795961

[ref7] Ravishankara A. R.; Hancock G.; Kawasaki M.; Matsumi Y. Photochemistry of Ozone: Surprises and Recent Lessons. Science 1998, 280, 60–61. 10.1126/science.280.5360.60.

[ref8] McBane G. C.; Nguyen L. T.; Schinke R. Photodissociation of ozone in the Hartley band: Product state and angular distributions. J. Chem. Phys. 2010, 133, 144312 10.1063/1.3491813. 20950005

[ref9] Picconi D.; Grebenshchikov S. Y. Signatures of a conical intersection in photofragment distributions and absorption spectra: Photodissociation in the Hartley band of ozone. J. Chem. Phys. 2014, 141, 074311 10.1063/1.4892919. 25149790

[ref10] Qu Z.-W.; Zhu H.; Grebenshchikov S. Y.; Schinke R. The photodissociation of ozone in the Hartley band: A theoretical analysis. J. Chem. Phys. 2005, 123, 074305 10.1063/1.2001650. 16229568

[ref11] Gunthardt C. E.; Aardema M. N.; Hall G. E.; North S. W. Evidence for lambda doublet propensity in the UV photodissociation of ozone. J. Chem. Phys. 2019, 151, 224302 10.1063/1.5131504. 31837678

[ref12] Grebenshchikov S. Y.; Rosenwaks S. Ab Initio Quantum Mechanical Study of the *O*(^1^*D*) Formation in the Photolysis of Ozone between 300 and 330 nm. J. Phys. Chem. A 2010, 114, 9809–9819. 10.1021/jp1028849. 20509638

[ref13] O’Keeffe P.; Ridley T.; Sheard H. A.; Lawley K. P.; Donovan R. J.; Lewis B. R. The *d*^1^Π_*g*_ (*v* = 1) Rydberg state of *O*_2_: Optical-optical double-resonance and Huggins-band ozone-photolysis, resonance-enhanced multiphoton-ionization studies with a *b*^1^Σ_*g*_^+^(*v* = 0)-state platform. J. Chem. Phys. 2002, 117, 8705–8709. 10.1063/1.1513462.

[ref14] Wine P. H.; Ravishankara A. R. *O*_3_ photolysis at 248 nm and *O*(^1^*D*_2_) quenching by *H*_2_*O*, *CH*_4_, *H*_2_, and *N*_2_*O*: *O*(^3^*P*_*J*_) yields. Chem. Phys. 1982, 69, 365–373. 10.1016/0301-0104(82)88075-0.

[ref15] Turnipseed A. A.; Vaghjiani G. L.; Gierczak T.; Thompson J. E.; Ravishankara A. R. The photochemistry of ozone at 193 and 222 nm. J. Chem. Phys. 1991, 95, 3244–3251. 10.1063/1.460881.

[ref16] Takahashi K.; Hayashi S.; Matsumi Y.; Taniguchi N.; Hayashida S. Quantum yields of *O*(^1^*D*) formation in the photolysis of ozone between 230 and 308 nm. J. Geophys. Res. 2002, 107, ACH 11–1–ACH 11–8.

[ref17] Talukdar R. K.; Longfellow C. A.; Gilles M. K.; Ravishankara A. R. Quantum yields of *O*(^1^*D*) in the photolysis of ozone between 289 and 329 nm as a function of temperature. Geophys. Res. Lett. 1998, 25, 143–146. 10.1029/97GL03354.

[ref18] Sparks R. K.; Carlson L. R.; Shobatake K.; Kowalczyk M. L.; Lee Y. T. Ozone photolysis: A determination of the electronic and vibrational state distributions of primary products. J. Chem. Phys. 1980, 72, 1401–1402. 10.1063/1.439205.

[ref19] Valentini J. J.; Gerrity D. P.; Phillips D. L.; Nieh J.-C.; Tabor K. D. CARS spectroscopy of *O*_2_(^1^Δ_*g*_) from the Hartley band photodissociation of *O*_3_: Dynamics of the dissociation. J. Chem. Phys. 1987, 86, 6745–6756. 10.1063/1.452374.

[ref20] Miller R. L.; Suits A. G.; Houston P. L.; Toumi R.; Mack J. A.; Wodtke A. M. The “Ozone Deficit” Problem: *O*_2_(*X*, *v* ≥ 26) + *O*(^3^*P*) from 226-nm Ozone Photodissociation. Science 1994, 265, 1831–1838. 10.1126/science.265.5180.1831. 17797220

[ref21] Leforestier C.; LeQuéré F.; Yamashita K.; Morokuma K. Theoretical study of the ultraviolet photodissociation of ozone. Comparison with experiments. J. Chem. Phys. 1994, 101, 3806–3818. 10.1063/1.467498.

[ref22] Thelen M.-A.; Gejo T.; Harrison J. A.; Huber J. R. Photodissociation of ozone in the Hartley band: Fluctuation of the vibrational state distribution in the *O*_2_(^1^Δ_*g*_) fragment. J. Chem. Phys. 1995, 103, 7946–7955. 10.1063/1.470212.

[ref23] Takahashi K.; Kishigami M.; Taniguchi N.; Matsumi Y.; Kawasaki M. Photofragment excitation spectrum for *O*(^1^*D*) from the photodissociation of jet-cooled ozone in the wavelength range 305–329 nm. J. Chem. Phys. 1997, 106, 6390–6397. 10.1063/1.473629.

[ref24] Denzer W.; Hancock G.; Pinot de Moira J. C.; Tyley P. L. Spin-forbidden dissociation of ozone in the Huggins bands. Chem. Phys. 1998, 231, 109–119. 10.1016/S0301-0104(97)00328-5.

[ref25] Geiser J. D.; Dylewski S. M.; Mueller J. A.; Wilson R. J.; Toumi R.; Houston P. L. The vibrational distribution of *O*_2_(*X*^3^Σ_*g*_^–^) produced in the photodissociation of ozone between 226 and 240 and at 266 nm. J. Chem. Phys. 2000, 112, 1279–1286. 10.1063/1.480679.

[ref26] Matsumi Y.; Kawasaki M. Photolysis of Atmospheric Ozone in the Ultraviolet Region. Chem. Rev. 2003, 103, 4767–4782. 10.1021/cr0205255. 14664632

[ref27] Hancock G.; Horrocks S. J.; Pearson P. J.; Ritchie G. A. D.; Tibbetts D. F. Photolysis wavelength dependence of the translational anisotropy and the angular momentum polarization of *O*_2_(*a*^1^Δ_*g*_) formed from the UV photodissociation of *O*_3_. J. Chem. Phys. 2005, 122, 244321 10.1063/1.1944719. 16035771

[ref28] Qu Z.-W.; Zhu H.; Grebenshchikov S. Y.; Schinke R. The triplet channel in the photodissociation of ozone in the Hartley band: Classical trajectory surface hopping analysis. J. Chem. Phys. 2005, 122, 191102 10.1063/1.1925608. 16161556

[ref29] Schinke R.; McBane G. C. Photodissociation of ozone in the Hartley band: Potential energy surfaces, nonadiabatic couplings, and singlet/triplet branching ratio. J. Chem. Phys. 2010, 132, 044305 10.1063/1.3299249. 20113031

[ref30] Warter M. L.; Gunthardt C. E.; Wei W.; McBane G. C.; North S. W. Nascent *O*_2_ (*a*^1^Δ_*g*_, *v* = 0, 1) rotational distributions from the photodissociation of jet-cooled *O*_3_ in the Hartley band. J. Chem. Phys. 2018, 149, 134309. 30292221 10.1063/1.5051540

[ref31] Aardema M. N.; McBane G. C.; North S. W. Ozone Photodissociation in the Singlet Channel at 226 nm. J. Phys. Chem. A 2022, 126, 6898–6907. 10.1021/acs.jpca.2c04832. 36129835

[ref32] Dylewski S. M.; Geiser J. D.; Houston P. L. The energy distribution, angular distribution, and alignment of the *O*(^1^*D*_2_) fragment from the photodissociation of ozone between 235 and 305 nm. J. Chem. Phys. 2001, 115, 7460–7473. 10.1063/1.1405439.

[ref33] Han S.; Gunthardt C. E.; Dawes R.; Xie D.; North S. W.; Guo H. Origin of the “odd” behavior in the ultraviolet photochemistry of ozone. Proc. Natl. Acad. Sci. U.S.A. 2020, 117, 21065–21069. 10.1073/pnas.2006070117. 32817468 PMC7474587

[ref34] O’Keeffe P.; Ridley T.; Wang S.; Lawley K. P.; Donovan R. J. Photodissociation of ozone between 335 and 352 nm to give *O*_2_(*b*^1^Σ_*g*_^+^) + *O*(^3^*P*_*J*_). Chem. Phys. Lett. 1998, 298, 368–374. 10.1016/S0009-2614(98)01190-7.

[ref35] Zhu H.; Qu Z.-W.; Tashiro M.; Schinke R. On spin-forbidden processes in the ultra-violet photodissociation of ozone. Chem. Phys. Lett. 2004, 384, 45–51. 10.1016/j.cplett.2003.11.095.

[ref36] Atkinson R.; Baulch D. L.; Cox R. A.; Hampson R. F. Jr.; Kerr J. A.; Rossi M. J.; Troe J. Evaluated Kinetic and Photochemical Data for Atmospheric Chemistry: Supplement VI. IUPAC Subcommittee on Gas Kinetic Data Evaluation for Atmospheric Chemistry. J. Phys. Chem. Ref. Data 1997, 26, 1329–1499. 10.1063/1.556010.

[ref37] Fairchild C. E.; Stone E. J.; Lawrence G. M. Photofragment spectroscopy of ozone in the uv region 270–310 nm and at 600 nm. J. Chem. Phys. 1978, 69, 3632–3638. 10.1063/1.437071.

[ref38] Sinha A.; Imre D.; Goble J. H. Jr.; Kinsey J. L. Excitation spectroscopy of jet-cooled ozone: The Huggins system. J. Chem. Phys. 1986, 84, 6108–6114. 10.1063/1.450801.

[ref39] Le Quéré F.; Leforestier C. Theoretical calculation of the Huggins band of ozone. Chem. Phys. Lett. 1992, 189, 537–541. 10.1016/0009-2614(92)85246-7.

[ref40] Bludsky O.; Jensen P. The calculation of the bound and quasibound vibrational states for ozone in its ^1^*B*_2_ electronic state. Mol. Phys. 1997, 91, 653–661. 10.1080/00268979709482755.

[ref41] Qu Z.-W.; Zhu H.; Tashiro M.; Schinke R.; Farantos S. C. The Huggins band of ozone: Unambiguous electronic and vibrational assignment. J. Chem. Phys. 2004, 120, 6811–6814. 10.1063/1.1711589. 15267579

[ref42] Qu Z.-W.; Zhu H.; Grebenshchikov S. Y.; Schinke R.; Farantos S. C. The Huggins band of ozone: A theoretical analysis. J. Chem. Phys. 2004, 121, 11731–11745. 10.1063/1.1814098. 15634138

[ref43] Zhu H.; Qu Z.-W.; Grebenshchikov S. Y.; Schinke R.; Malicet J.; Brion J.; Daumont D. The Huggins band of ozone: Assignment of hot bands. J. Chem. Phys. 2005, 122, 024310 10.1063/1.1825380. 15638589

[ref44] Kim H.; Park J.; Niday T. C.; North S. W. The UV photodissociation dynamics of ClO radical using velocity map ion imaging. J. Chem. Phys. 2005, 123, 174303 10.1063/1.2083487. 16375524

[ref45] Grubb M. P.; Warter M. L.; Johnson K. M.; North S. W. Ion Imaging Study of *NO*_3_ Radical Photodissociation Dynamics: Characterization of Multiple Reaction Pathways. J. Phys. Chem. A 2011, 115, 3218–3226. 10.1021/jp200110e. 21446659

[ref46] Schmaunz A.; Kensy U.; Slenczka A.; Dick B. Velocity resolved REMPI spectroscopy: a new approach to the study of photodissociation dynamics. Phys. Chem. Chem. Phys. 2009, 11, 7115–7119. 10.1039/b909037a. 19672518

[ref47] Wenge A. M.; Schmaunz A.; Kensy U.; Dick B. Photodissociation dynamics of tert-butylnitrite following excitation to the S_1_ and S_2_ states. A study by velocity-map ion-imaging and 3D-REMPI spectroscopy. Phys. Chem. Chem. Phys. 2012, 14, 7076–7089. 10.1039/c2cp40349h. 22491099

[ref48] Lee K. L. K.; Quinn M. S.; Maccarone A. T.; Nauta K.; Houston P. L.; Reid S. A.; Jordan M. J. T.; Kable S. H. Two roaming pathways in the photolysis of CH_3_CHO between 328 and 308 nm. Chem. Sci. 2014, 5, 4633–4638. 10.1039/C4SC02266A.

[ref49] Quinn M. S.; Andrews D. U.; Nauta K.; Jordan M. J. T.; Kable S. H. The energy dependence of CO(v,J) produced from H_2_CO via the transition state, roaming, and triple fragmentation channels. J. Chem. Phys. 2017, 147, 013935 10.1063/1.4983138. 28688440

[ref50] Luque J.; Crosley D. LIFBASE: Database and Spectral Simulation Program (v 1.6). SRI International Report MP 1999, 99–009.

[ref51] Wei W.; Wallace C. J.; Grubb M. P.; North S. W. A method of extracting speed-dependent vector correlations from 2 + 1 REMPI ion images. J. Chem. Phys. 2017, 147, 013947 10.1063/1.4985704. 28688436

[ref52] Dixon R. N. The determination of the vector correlation between photofragment rotational and translational motions from the analysis of Doppler-broadened spectral line profiles. J. Chem. Phys. 1986, 85, 1866–1879. 10.1063/1.451131.

[ref53] Morrill J. S.; Ginter M. L.; Hwang E. S.; Slanger T. G.; Copeland R. A.; Lewis B. R.; Gibson S. T. Two-photon REMPI spectra from *a*^1^Δ_*g*_ and *b*^1^Σ_*g*_^+^ to *d*^1^Π_*g*_ in *O*_2_. J. Mol. Spectrosc. 2003, 219, 200–216. 10.1016/S0022-2852(03)00074-2.

[ref54] Bray R. G.; Hochstrasser R. M. Two-photon absorption by rotating diatomic molecules. Mol. Phys. 1976, 31, 1199–1211. 10.1080/00268977600100931.

[ref55] Le Roy R. J.; Kraemer G. T. BCONT 2.2 A Computer Program for Calculating Bound → Continuum Transition Intensities for Diatomic Molecules Univ. Waterloo Chem. Phys. Res. Report CP-650R^2^ 2004.

[ref56] Morrill J. S.; Ginter M. L.; Lewis B. R.; Gibson S. T. The (*X*^2^Π_*g*_)*nsσ*_*g*_^1,3^Π_*g*_ Rydberg states of *O*_2_: Spectra, structures, and interactions. J. Chem. Phys. 1999, 111, 173–185. 10.1063/1.479264.

