# Supplementary material for: Rotational Distributions and Imaging of Singlet O2 Following Spin-Forbidden Photodissociation of O3
Source: J Phys Chem A. 2023 Aug 4;127(34):7101–14. doi: 10.1021/acs.jpca.3c02736 (PMC10863062; doi:10.1021/acs.jpca.3c02736)
Supplement: Supplementary file 1 — jp3c02736_si_001.pdf [file jp3c02736_si_001.pdf]

Rotational Distributions and Imaging of Singlet O_2 following Spin-Forbidden Photodissociation of O_3

Supporting Information

Megan N. Aardema, Megan Fast, Benjamen Meas, Simon W. North*

Department of Chemistry, Texas A&M University, College Station Texas 77842

1 Temperature Calibration

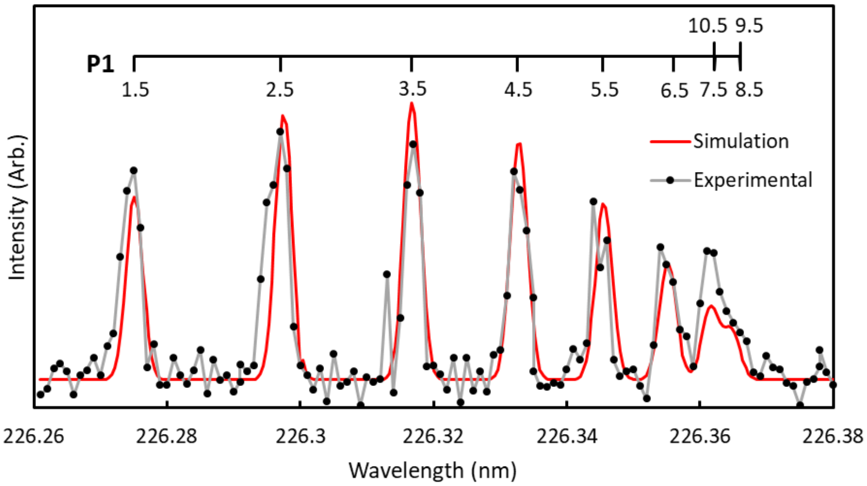

Figure S1: REMPI spectrum of NO probed via the $A \leftarrow X$ transition near 226 nm. The black circles are the experimental spectrum and the red line is a simulated 50 K distribution used to fit the experimental distribution.

The temperature of the molecular beam was measured by collecting a REMPI spectrum of NO with the same running conditions as were used for O_3 experiments and a vapor pressure similar to the vapor pressure of O_3 on silica beads in a -40°C bath, resulting in a similar seeding ratio. NO was probed with 1+1 REMPI at 226 nm via the $A \leftarrow X$ transition. Because the NO was primarily in the center of the image, the ion optics were defocused and

low laser power (50-100 μJ) was used to spread out the signal on the detector and avoid saturation. Figure S1 shows the experimental NO REMPI indicated by black circles and a 50 K distribution simulated with LIFBASE.[1]

2 Estimating the $^3A'$ and $^3A''$ Crossing Probability with the B State

To estimate the probability of transitions from the B state to $^3A'$ and $^3A''$ states, a 1D Landau-Zener model was used. The transition probability P for an individual state can be calculated with Equation 1.

$$P = 2\ exp\left[\frac{-2\pi\Delta_T^2}{\hbar\nu|F_B - F_T|}\right]\left(1 - exp\left[\frac{-2\pi\Delta_T^2}{\hbar\nu|F_B - F_T|}\right]\right) \tag{1}$$

In this equation, Δ_T is the spin-orbit matrix element between the B and triplet states reported by Grebenshchikov and Rosenwaks,[2] ν is the relative velocity and F_B and F_T are the slopes of the B and triplet state potentials, respectively, at the crossing point. The relative velocity was estimated by subtracting the energy of the crossing point from the photon excitation energy and converting the energy to fragment velocity. The potential energy curves reported by Grebenshchikov and Rosenwaks were used to determine the slopes of the potentials at the crossing points. The relative velocities and resulting crossing probabilities are shown in Table 1. The triplet states are numbered by increasing energy and the number in parentheses indicates the first, second, or third component of the triplet state. There are additional $^3A'$ and $^3A''$ states that cross the B state but only the states correlating with $O_2(a^1\Delta_g)$ and $O(^3P)$ products were included. Summing the probabilities of transition to all $^3A'$ states and all $^3A''$ states should give the relative intensity expected for even and odd rotational states in the REMPI spectrum.

$^3A'$ State	Coupling	Relative Velocity	Transition Probability
3 $^3A'(1)$	30 cm^{-1}	1470 m/s	4.0x10^{-3}
3 $^3A'(3)$	30 cm^{-1}	1470 m/s	4.0x10^{-3}
4 $^3A'(1)$	19 cm^{-1}	2038 m/s	8.0x10^{-4}
4 $^3A'(3)$	19 cm^{-1}	2038 m/s	8.0x10^{-4}
5 $^3A'(1)$	20 cm^{-1}	1922 m/s	7.3x10^{-4}
5 $^3A'(3)$	20 cm^{-1}	1922 m/s	7.3x10^{-4}
$^3A''$ State	Coupling	Relative Velocity	Transition Probability
3 $^3A''(1)$	16 cm^{-1}	2020 m/s	1.0x10^{-3}
3 $^3A''(2)$	52 cm^{-1}	2020 m/s	1.1x10^{-2}
3 $^3A''(3)$	16 cm^{-1}	2020 m/s	1.0x10^{-3}
4 $^3A''(1)$	11 cm^{-1}	2055 m/s	3.5x10^{-4}
4 $^3A''(2)$	37 cm^{-1}	2055 m/s	3.9x10^{-3}
4 $^3A''(3)$	11 cm^{-1}	2055 m/s	3.5x10^{-4}

Table 1: The triplet states are numbered by increasing energy, and the number in parentheses corresponds to the first, second, or third component of the triplet state. The spin-orbit matrix elements from Grebenshchikov and Rosenwaks are shown in the second column.[2] The relative velocity is calculated by subtracting the potential energy at the crossing point from the excitation energy (320 nm) and converting to velocity. The transition probability is calculated using Equation 1.

3 O_2 Ion Images following O_3 Dissociation near 320 nm

Figure S2 shows the outer edge of reconstructed ion images of O_2 following photodissociation of O_3 near 320 nm. The images were collected at wavelengths corresponding to R- and S-branch transitions of $O_2(a^1\Delta_g)$ in rotational states j=17-20 probed via the 2-0 band of the $O_2(d^1\Pi_g \leftarrow\leftarrow a^1\Delta_g)$ transition. The images were collected in a 1-color experiment with a vertically polarized laser. The outer ring of the images corresponds to fragments formed in the spin-forbidden dissociation channel producing $O_2(a^1\Delta_g)$ and $O(^3P)$, and in several images the outer ring is split, indicating overlapping transitions of fragments in different rotational states. The speed distributions corresponding to the outer ring are shown on the right in Figure S2. The outer ring in the images corresponding to S-branch transitions of j=19 and 20 and the R-branch image of j=20 are unimodal, indicating fragments traveling a single speed. The speed distributions of the outer ring in the S-branch images of j=17 and 18 and the R-branch images of j=17, 18, and 19 have additional peaks at slower speeds, corresponding to higher rotational states. The difference in speeds between the peaks is consistent with the overlapping transitions assigned by Morrill et al.[3] in their 2-0 $O_2(d^1\Pi_g \leftarrow\leftarrow a^1\Delta_g)$ REMPI spectrum at 320 nm. Signal from the O-branch transition of

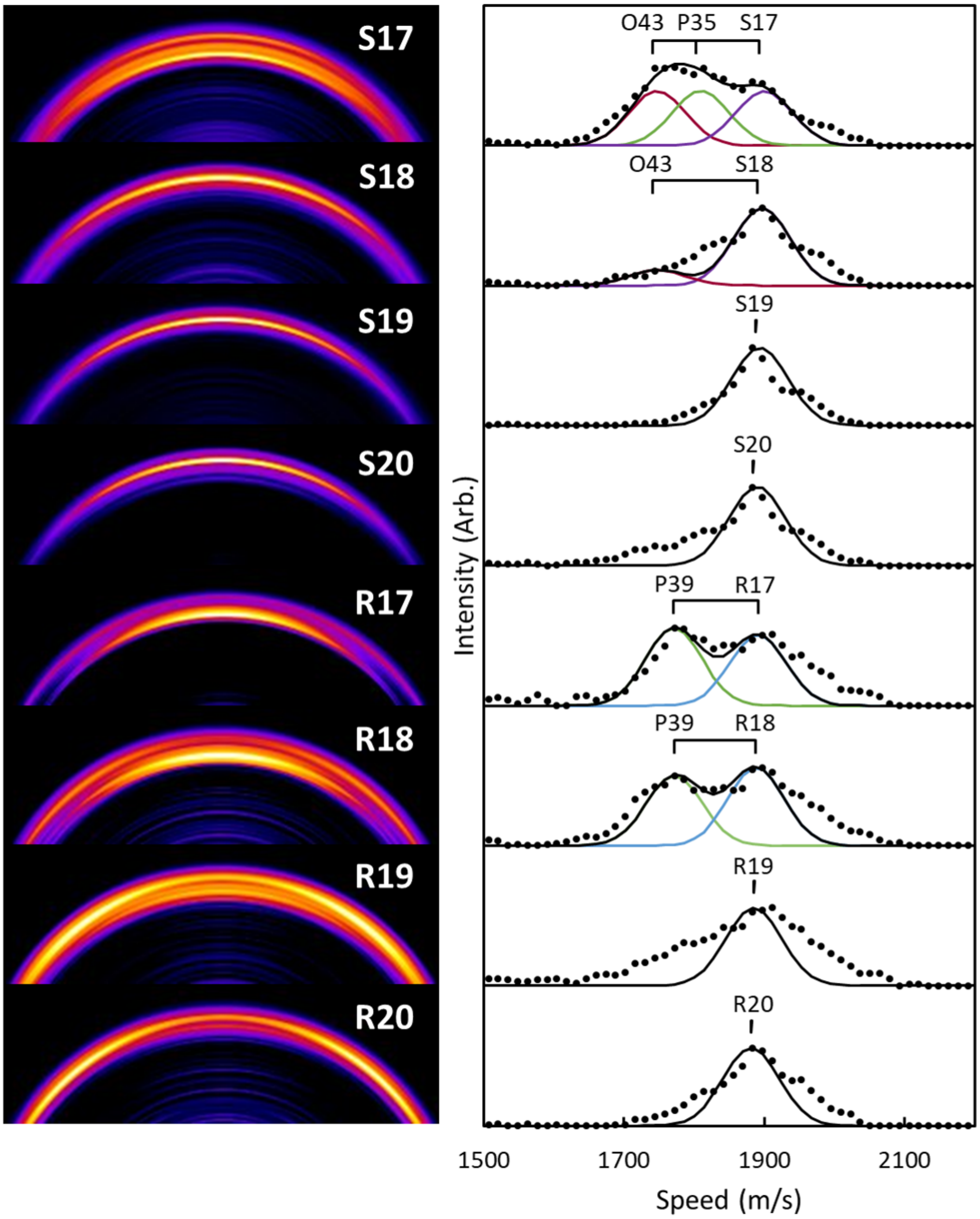

Figure S2: The outer edge of reconstructed $O_2(a^1\Delta_g, v = 0)$ ion images collected at wavelengths corresponding to S- and R-branch transitions of j=17-20 in a 1-color experiment with a 320 nm vertically polarized laser are shown on the left. The outer ring in each image is due to the spin-forbidden dissociation channel, and the corresponding speed distributions for the outer edge of each image are shown on the right. The speed distributions are fit with Gaussian distributions centered at the speeds expected for fragments in the indicated rotational states.

j=43 is minor in the S-branch image of j=18. The R-branch image of j=19 appears to have two rings in the image, which may indicate the presence of a yet unassigned transition of an O-branch transition near j=40-42, which would be consistent with the 2-fold symmetry observed for the slower fragments. An O-branch transition may also account for the 2-fold symmetry observed for the slower fragments in the R-branch j=17 and 18 images, which is inconsistent with their P-branch assignments. The speed distributions are fit with Gaussian distributions centered at the speeds expected for fragments in the given rotational state, with σ=40 m/s, corresponding to approximately 2% uncertainty in velocity.

4 Scaling Peak Intensities for $O_2(d^1\Pi_g)$

Because the lower vibrational states of the $O_2(d^1\Pi_g)$ state are perturbed, fitting REMPI spectra that access these perturbed vibrational levels is difficult. The perturbations cause deviations in both peak position and peak intensity in the observed spectrum. The peak positions have been previously reported by Morrill et al. and O'Keeffe et al., and O'Keeffe et al. extracted rotational state populations of $O_2(b^1\Sigma_g^+, v = 0)$ by correcting for the perturbations.[3,4] Perturbations in the resonant state of the REMPI scheme should affect all transitions that lead to the same rotational level in the upper electronic state equally. To fit the 330 nm $O_2(d^1\Pi_g, v = 1 \leftarrow\leftarrow a^1\Delta_g, v = 0)$ REMPI spectrum, we used the 1-0 REMPI spectrum reported by Morrill et al. to estimate scaling factors for each rotational level of $O_2(d^1\Pi_g, v = 1)$ and used these scaling factors in the fit of our 330 nm REMPI spectrum.

Figure S3 shows the REMPI spectrum reported by Morrill et al. for the 280 nm dissociation of O_3, probed via the 1-0 band of the $O_2(d^1\Pi_g \leftarrow\leftarrow a^1\Delta_g)$ transition, along with our fits to the reported spectrum.[3] In the experiment, the authors used 46 mTorr O_3 in 7 Torr He, so the distribution of O_3 is expected to be thermalized. The spectrum was fit with the O_2 rotational state populations predicted by classical trajectory calculations for a 282 nm dissociation, depolarized by the classical model of Gunthardt et al. which broadens the distribution based on the rotational temperature of the parent molecules (assumed to be 300

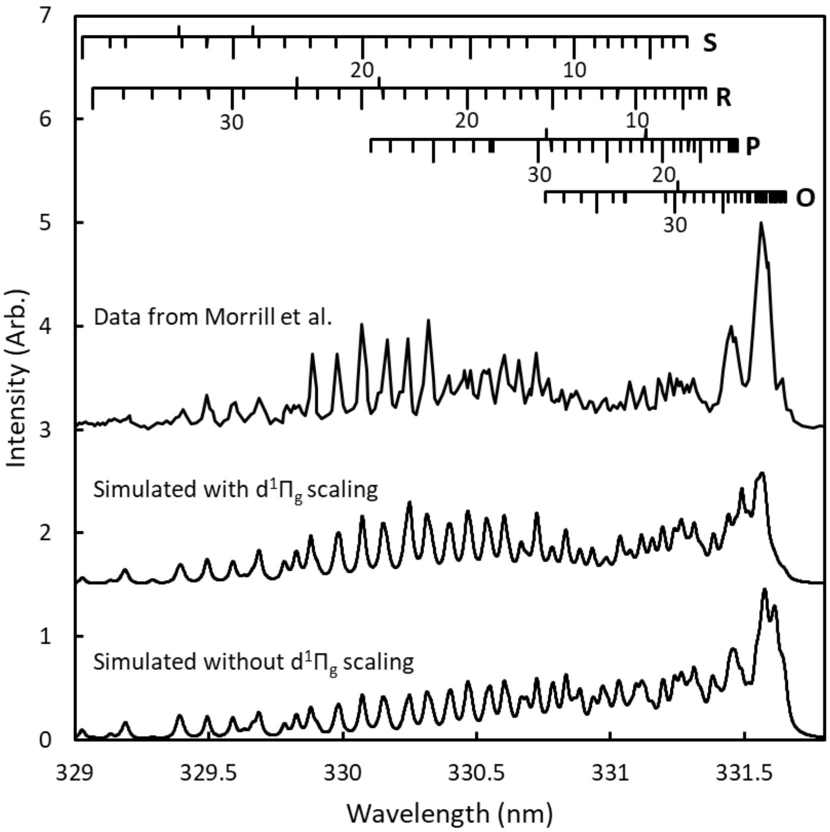

Figure S3: The top spectrum is the $O_2(d^1\Pi_g, v = 1 \leftarrow\leftarrow a^1\Delta_g, v = 0)$ reported by Morrill et al. following the 280 nm dissociation of O_3.[3] The middle spectrum is the fit to the experimental spectrum using depolarized O_2 rotational state populations calculated for a 282 nm dissociation of O_3 with intensities scaled based on the rotational state of the resonant $d^1\Pi_g$ state. The bottom spectrum is the fit to the experimental data with the same populations as above but without scaling intensities based on the $d^1\Pi_g$ state.

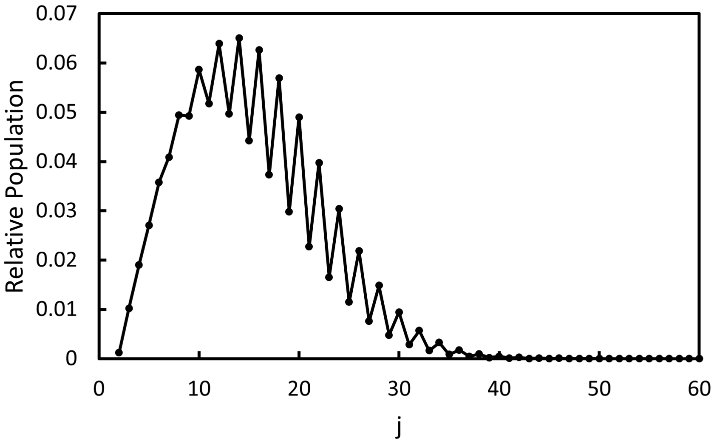

Figure S4: $O_2(a^1\Delta_g)$ rotational state populations calculated for a 282 nm dissociation of O_3 by classical trajectory calculations[5] and depolarized following the classical model of Gunthardt et al.[6] These populations were used to fit the spectrum in Figure S3.

K) and includes the even-odd alternation observed in $O_2(a^1\Delta_g)$ fragments.[5,6] The rotational state population used to fit the spectrum is shown in Figure S4.

Each rotational state of the $d^1\Pi_g$ state was scaled individually in the fitting of the spectrum, which affects transitions from each rotational branch that lead to the same resonant state. The top spectrum in Figure S3 is the experimental spectrum from Morrill et al. The middle spectrum is our fit to the experimental spectrum, using the depolarized rotational state distribution calculated for a 282 nm dissociation with scaling factors for each rotational state of $O_2(d^1\Pi_g)$ included. The bottom spectrum is fit with the same populations as the middle spectrum, but no scaling of $O_2(d^1\Pi_g)$ state. It is clear that including the scaling factors improves the fit to the experimental spectrum. The increase in intensity near 329.75 nm and the decrease in intensity near 331 nm are both better reproduced in the fit with the scaling factors than the fit without $d^1\Pi_g$ state scaling. The inclusion of these scaling factors should therefore improve the fit to the 1-0 REMPI of the $O_2(a^1\Delta_g)$ following 330 nm dissociation as well.

5 Rotational Distributions of $O_2(a^1\Delta_g, v = 0)$ and $O_2(b^1\Sigma_g^+, v = 0)$

In their analysis of $O(^3P_2)$ speed distributions extracted from ion images, Ulrich et al. assumed a single rotational temperature for all three electronic states of O_2 and all vibrational levels.[7] The authors fit the speed distributions both with a "full" model that allowed population of all energetically accessible vibrational levels of each electronic state, and a "restricted" model that limited population to the lower vibrational states. The full model utilized a rotational temperature of 1880 K, and the restricted model used 3660 K. The authors suggested 2500 K as an average temperature. Figure S5 shows a Boltzmann distribution for 1800, 2500, and 3660 K shown in blue, green, and purple, respectively, for both the $a^1\Delta_g$ and $b^1\Sigma_g$ states.

The distributions shown in black are the fits to the experimental spectra for $O_2(a^1\Delta_g, v = 0$ and $b^1\Sigma_g^+, v = 0)$ on the left and right, respectively. It is clear that the experimental

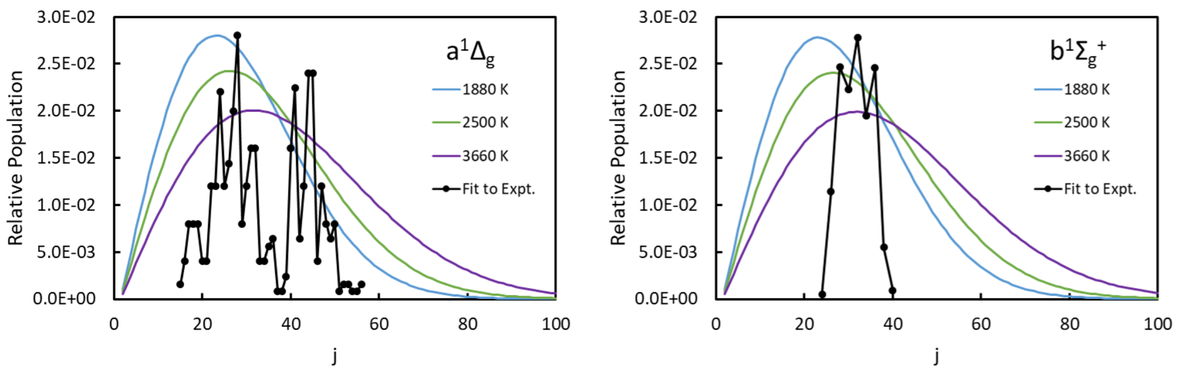

Figure S5: Comparison of the experimental rotational distributions of O_2 in both the $a^1\Delta_g$ (left) and $b^1\Sigma_g^+$ (right) states to Boltzmann distributions at 1880 K, 2500 K, and 3660 K. The black circles are the experimental distributions, and the blue, green, and purple curves represent distributions at 1880, 2500, and 3660 K, respectively.

distributions are both non-Boltzmann, and the Boltzmann distributions are broader than the experimental distributions. Furthermore, the rotational distributions of $O_2(a^1\Delta_g)$ and $O_2(b^1\Sigma_g^+)$ are significantly different from each other. This indicates that the two distributions cannot be described by a single temperature. The rotational distribution of $O_2(a^1\Delta_g)$ used in fitting the $O_2(d^1\Pi_g, v = 1 \leftarrow\leftarrow a^1\Delta_g, v = 0)$ REMPI spectrum is bimodal, but the R- and S-branch transitions of the rotational states with low population in the middle of the distribution correspond to the wavelength region where no experimental spectrum was collected. The O- and P-branch transitions of these states overlap the lower rotational states in the R and S branches. The lack of data in the middle of the spectrum, along with the assumptions included in scaling the rotational levels of the resonant $d^1\Pi_g$ state make determining the relative populations of the rotational states difficult, but the rotational distribution is clearly broad.

The rotational distribution of $O_2(b^1\Sigma_g^+)$ is narrow relative to both the experimental $O_2(a^1\Delta_g)$ distribution and the Boltzmann distributions for all three temperatures reported by Ulrich et al. For additional comparison, REMPI spectra were simulated for the 4-0 band of the $O_2(d^1\Pi_g \leftarrow\leftarrow b^1\Sigma_g^+)$ transition with a Boltzmann rotational state distribution. The same spectroscopic constants and linewidth trend used for the experimental fit were used in this simulation. Figure S6 shows the simulations of the REMPI spectra for 1880 K (left)

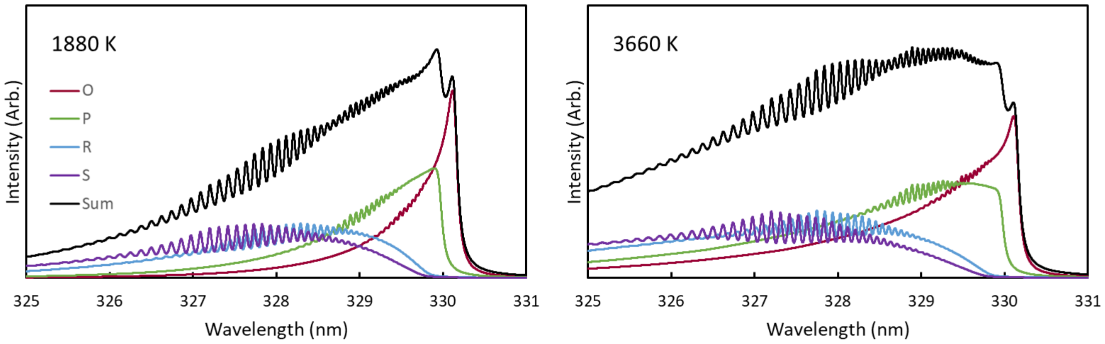

Figure S6: Simulated REMPI spectra for O_2 with a Boltzmann rotational distribution at 1880 K on the left and 3660 K on the right. The simulation utilizes the $O_2(d^1\Pi_g, v = 4 \leftarrow\leftarrow b^1\Sigma_g^+, v = 0)$ REMPI scheme near 330 nm. The maroon, green, blue and purple lines represent the O, P, R, and S branches, and the sum of the branches is shown in black.

and 3660 K (right). In each plot, the O, P, R, and S branches are shown in maroon, green, blue, and purple, respectively, and the sum of the branches is shown in black. The simulated spectra are considerably broader than the experimental spectra with a large amount of signal below the structured region due to the overlap of the branches. This is inconsistent with the small number of well-resolved peaks in the experimental spectrum.

6 REMPI spectra of v=1-4 of $O_2(b^1\Sigma_g^+)$

Figure S7 shows the 2D-REMPI spectrum of O_2 following O_3 dissociation near 330 nm collected in a 1-color experiment. An ion image was collected at each wavelength step, symmetrized, and reconstructed to obtain a radial distribution at each wavelength, which was then converted to a speed distribution. Each horizontal band of signal corresponds to fragments traveling a different speed and can be assigned to an electronic and vibrational state of the O_2 fragment. The spectrum is integrated over a narrow range of speeds to obtain rotational spectra of each electronic and vibrational state shown on the right. The fragments with the fastest speed correspond to $O_2(a^1\Delta_g, v = 0)$, and the additional bands are assigned to different vibrational levels of $O_2(b^1\Sigma_g^+)$, based on the speed distribution. The regions of integration are indicated by the white lines on the spectrum in Figure S7, and the corresponding rotational spectrum for each region is shown on the right, along

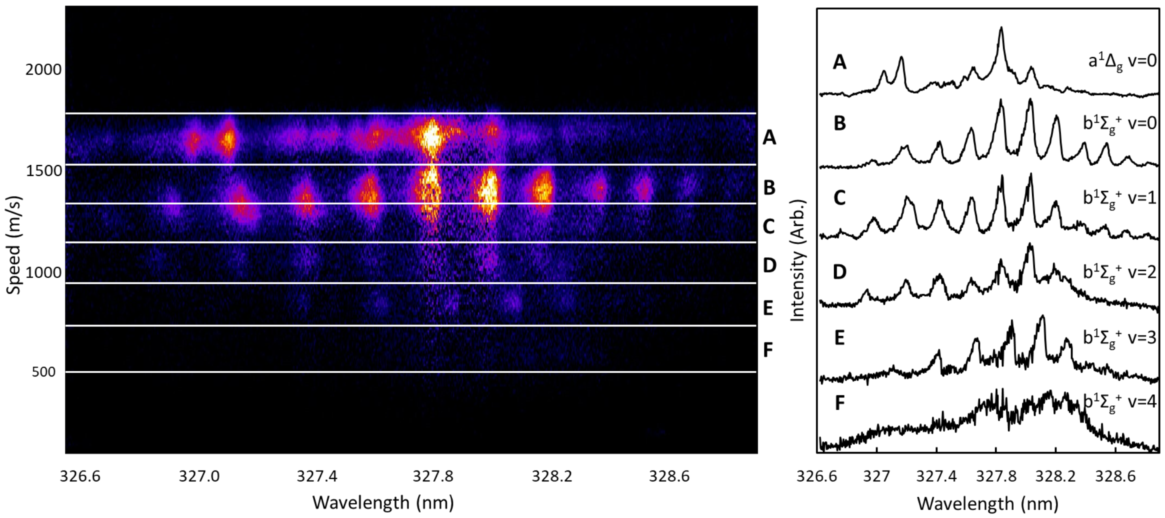

Figure S7: The 2D-REMPI spectrum of O_2 was collected in a 1-color experiment following the dissociation of O_3 near 330 nm. Images were collected at each wavelength step, symmetrized, and reconstructed. The resulting speed distribution is plotted as a function of wavelength. Each horizontal band in the spectrum indicates a different electronic or vibrational state of O_2. Fragments at the fastest speeds are assigned to v=0 of the $a^1\Delta_g$ state, and the other bands are v=0-4 of the $b^1\Sigma_g^+$ state. The white lines indicate regions that are integrated to obtain the 1D rotational spectra shown on the right.

with the assigned electronic and vibrational state. The fits to the rotational spectra for $O_2(a^1\Delta_g, v = 0)$ and $O_2(b^1\Sigma_g^+, v = 0, 1$ and $4)$ are discussed in the main paper.

The fits to the rotational spectra for $O_2(b^1\Sigma_g^+, v = 1 - 4)$, corresponding to regions C-F in Figure S7, are shown in Figure S8. The ground vibrational state of $O_2(b^1\Sigma_g^+)$ is probed via v=4 of the resonant $d^1\Pi_g$ state near 330 nm, so the REMPI schemes of v=1, 2, 3, and 4 of the $b^1\Sigma_g^+$ state should be excited to v=5, 6, 7, and 8 of the $d^1\Pi_g$ state to require similar transition energies. The experimental data for each spectrum is indicated by black circles. The O, P, R, and S branches are shown in maroon, green, blue and purple, with the sum of the branches shown in black. The rotational state assignments are indicated by combs at the top. The corresponding rotational state populations for each vibrational level are shown on the right, and the j-dependent linewidths used in the simulated spectra are reported in Table 3.

Although the $O_2(b^1\Sigma_g^+, v = 4)$ signal is difficult to see by eye in Figure S7, the speed distribution of the 2D-REMPI spectrum indicates population of the v=4 vibrational level. In comparison to the lower vibrational states, the rotational spectrum for v=4 is less structured,

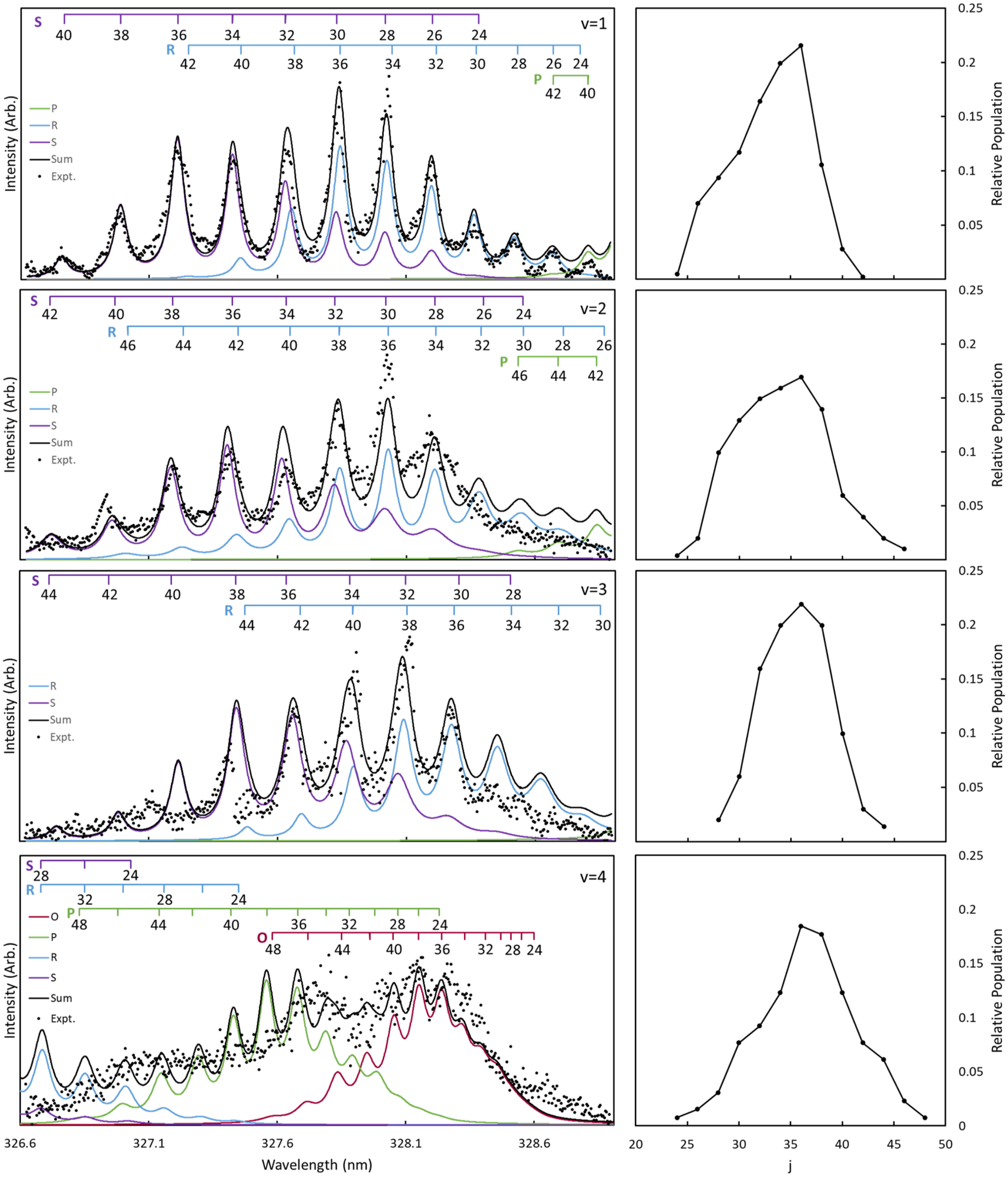

Figure S8: Rotational spectra obtained by integrating the 2D-REMPI in Figure S7 over ranges of speeds corresponding to regions C, D, E, and F. The experimental data is shown in black circles for $O_2(b^1\Sigma_g^+)$ in v=1, v=2, v=3, and v=4 from top to bottom along with the rotational state distributions used to fit each spectrum. The simulated O, P, R, and S branches are shown in maroon, green, blue, and purple, respectively, with the sum of the branches shown by the solid black line.

likely due in part to low signal. The peaks in the v=4 spectrum are much closer together than the peaks in the lower vibrational states, suggesting O- and P-branch transitions, rather than R- and S-branch transitions assigned to the v=0-3 spectra.

Spectroscopic constants for $O_2(b^1\Sigma_g^+, v = 1$, and 2) were obtained by fitting term values reported by Morrill et al.[3] The spectroscopic constants for v=3 and 4 were estimated from the constants for v=0-2. The lower vibrational levels (v=0-4) of the $d^1\Pi_g$ state have previously been used in REMPI schemes and the rotational state energies have been assigned,[3,4] but because vibrational states v=0-3 are highly perturbed, the spectroscopic constants of vibrational states v=5-7 cannot be estimated from lower vibrational levels. The vibrational energy levels have been previously calculated and estimated from kinetic energy release spectra,[8–10] but the rotational constants have not been reported to our knowledge. Rotational constants for v=5-8 were calculated using the BCONT program which determines the eigenvalues for a potential.[11] The potential calculated by Li et al.[12] was adjusted to fit the vibrational levels calculated by Morrill et al.,[8] and the rotational constants were calculated for the adjusted potential. The calculated rotational constant for v=4 was slightly different than the constant obtained by fitting experimental spectra, so the the b constants for v=5-8 were scaled by the same amount required for v=4 to account for this difference.

The vibrational levels of the $d^1\Pi_g$ state needed to be shifted to lower energies relative to previous calculations and kinetic energy release experiments to fit the measured spectra. The energy of v=5 was shifted by 330 cm^{-1}, v=6 by 690 cm^{-1}, and v=7 by 1110 cm^{-1} relative to calculations from Morrill et al.[8] Because the spectrum of the 8-4 transition was fit with O- and P-branch transitions rather than R- and S-branch transitions, the required energy shift was less than expected based on the shifts of the lower vibrational levels. The energy of v=8 was shifted by 1130 cm^{-1}. Despite these shifts, we believe the assignments of the 5-1, 6-2, 7-3, and 8-4 bands of the $O_2(d^1\Pi_g \leftarrow\leftarrow b^1\Sigma_g^+)$ transition are the most reasonable for the observed spectra. The spacing of the peaks in the rotational spectra are consistent with the expected spacing of the R- and S-branch transitions for v=0-3, and the number of peaks indicates significant overlap between peaks in the two branches. There is no evidence

State	T (cm^{-1})	b (cm^{-1})	d (cm^{-1})
$O_2(b^1\Sigma_g^+, v=0)$	13122.32	1.39107	5.25865x10^{-6}
$O_2(b^1\Sigma_g^+, v=1)$	14527.0	1.37301	5.43777x10^{-6}
$O_2(b^1\Sigma_g^+, v=2)$	15903.7	1.35465	5.51256x10^{-6}
$O_2(b^1\Sigma_g^+, v=3)$	17252.07	1.33649	5.48302x10^{-6}
$O_2(b^1\Sigma_g^+, v=4)$	18572.85	1.31828	5.34915x10^{-6}
$O_2(d^1\Pi_g, v=4)$	73746.87	1.588308	4.7023x10^{-6}
$O_2(d^1\Pi_g, v=5)$	75157.58	1.56724	5.09413x10^{-6}
$O_2(d^1\Pi_g, v=6)$	76503.31	1.54713	5.14598x10^{-6}
$O_2(d^1\Pi_g, v=7)$	77810.58	1.52689	5.20024x10^{-6}
$O_2(d^1\Pi_g, v=8)$	79465.00	1.50653	5.25708x10^{-6}

Table 2: Spectroscopic constants for $O_2(b^1\Sigma_g^+, v = 0-4)$ and $O_2(d^1\Pi_g, v = 4-8)$ used to fit the REMPI spectra are shown.

	Linewidth (cm^{-1})				
j($d^1\Pi_g$)	v=4	v=5	v=6	v=7	v=8
22					18
23					18
24					18
25	15.5	18	41		18
26	15	17	39.5		18
27	14.5	16	37		17.75
28	14	15	34		17.5
29	13.5	14	31	28	17.25
30	13	13	29	28	17
31	12.5	12	26	27	16.75
32	12	12	23	26	16.5
33	12	12	20	24	16.25
34	12	12	18	22	15.75
35	12	12	16	20	15.25
36	12	12	14.5	19	14.75
37	12	12	14	18	14.25
38	12	12	14	17	14
39	13	12	14.5	16	14
40	14	12	15	15	14.5
41	15.5	12	16	14	15
42	17	12	17	13.5	15.5
43		12	18	13	16
44		12	19	12.5	17
45			20	12	18
46			21	12	20
47			22		22
48			23		

Table 3: Linewidths used in fitting the REMPI spectra of the 4-0, 5-1, 6-2, 7-3, and 8-4 bands of the $O_2(d^1\Pi_g \leftarrow\leftarrow b^1\Sigma_g^+)$ transition shown in Figure 9 in the main paper and Figure S8.

of perturbations or suppressed odd rotational states, which would be indicative of a higher vibrational state of the $O_2(a^1\Delta_g)$ state, and the vibrational level shifts are too large to be

accounted for by excitation to the $C^3\Pi_g$ instead of the $d^1\Pi_g$ state. The constants used in the simulated spectra are shown in Table 2.

The simulations used to fit the spectra in Figure S8 also included two-photon line strengths from Bray and Hochstrasser[13] and j-dependent linewidths, reported in Table 3. The rotational state distributions for all vibrational levels are very similar, peaking near j=34 or 36, and extending from j∼24 to j∼44.

7 Comparison of REMPI Spectra with Huggins Band Structure

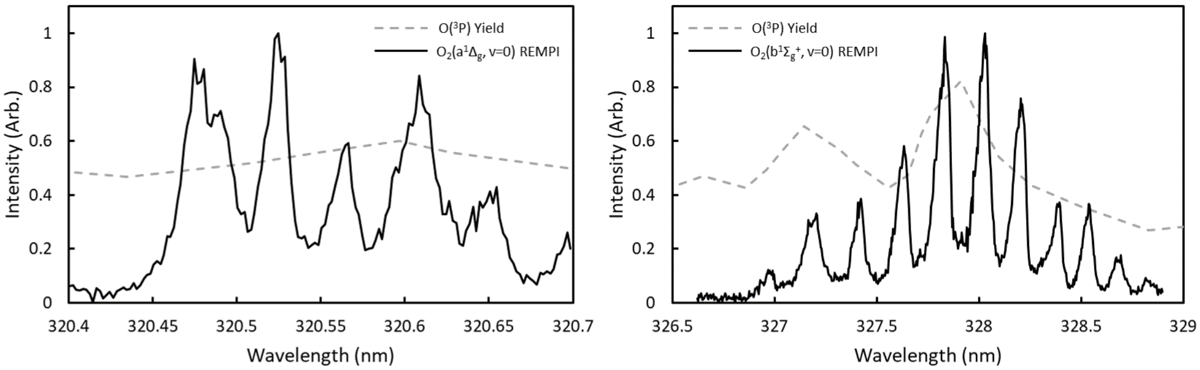

Figure S9: The $O_2(d^1\Pi_g, v = 2 \leftarrow\leftarrow a^1\Delta_g, v = 0)$ REMPI spectrum is shown on the left and the $O_2(d^1\Pi_g, v = 4 \leftarrow\leftarrow b^1\Sigma_g^+, v = 0)$ REMPI spectrum is shown on the right in black. The gray dashed lines show the $O(^3P)$ yield spectrum from O'Keeffe et al., representing the O_3 absorbance in the Huggins band.[14,15]

Figure S9 shows a comparison of the REMPI spectrum of $O_2(d^1\Pi_g, v = 2 \leftarrow\leftarrow a^1\Delta_g, v = 0)$ on the left and $O_2(d^1\Pi_g, v = 4 \leftarrow\leftarrow b^1\Sigma_g^+, v = 0)$ on the right with the $O(^3P)$ yield spectrum shown in gray. The $O(^3P)$ yield spectrum from O'Keeffe et al. is used to represent the absorption spectrum for a jet-cooled molecular beam, because the peaks should be sharper than a typical absorption spectrum collected between 200 and 300 K.[14,15] There is no clear correlation between the Huggins band structure and peak intensity in the $O_2(a^1\Delta_g)$ spectrum. While one of the absorption peaks overlaps with the high intensity region of the $O_2(b^1\Sigma_g^+)$ spectrum, the REMPI spectrum lacks a similar increase in intensity at the absorption peak near 327.2 nm and the REMPI signal decreases rapidly at short wavelengths while there is an additional absorption peak near 326.7 nm. Based on this comparison, we believe

the relative intensities of the REMPI peaks primarily reflect the rotational state populations rather than an increase in initial absorption of the O_3 parent.

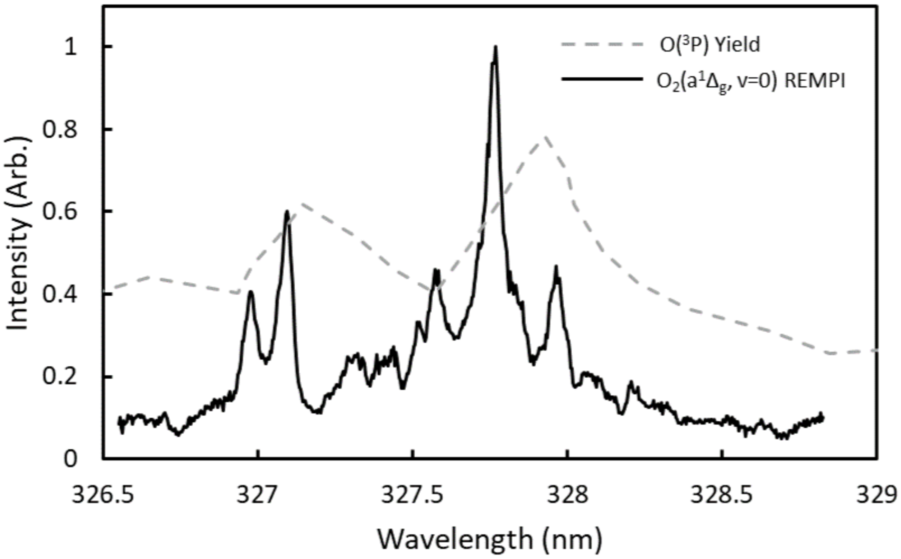

Figure S10: The $O_2(d^1\Pi_g, v = 1 \leftarrow\leftarrow a^1\Delta_g, v = 0)$ REMPI spectrum is shown in black and the $O(^3P)$ yield spectrum from O'Keeffe et al. representing the O_3 absorbance is represented by the gray dashed line.[14,15]

The high intensity peaks in the $O_2(d^1\Pi_g, v = 1 \leftarrow\leftarrow a^1\Delta_g, v = 0)$ spectrum from the 2D REMPI are consistent with Huggins band absorption peaks. This may indicate that while the $O_2(b^1\Sigma_g^+)$ production does not change significantly when dissociation occurs on or off an absorbance peak, the $O_2(a^1\Delta_g)$ production does appear to depend on the O_3 absorption. This would result in wavelength-dependent branching ratios, which may also depend on the vibrational modes excited in the initial O_3 absorption. Additional 2-color experiments in which dissociation occurs at a single wavelength are necessary to study this effect.

References

(1) Luque, J., and Crosley, D. (1999). LIFBASE: Database and Spectral Simulation Program (v 1.6). *SRI International Report MP 99-009.*

(2) Grebenshchikov, S. Y., and Rosenwaks, S. (2010). Ab Initio Quantum Mechanical Study of the $O(^1D)$ Formation in the Photolysis of Ozone between 300 and 330 nm. *J. Phys. Chem. A 114*, 9809–9819.

(*3*) Morrill, J. S., Ginter, M. L., Hwang, E. S., Slanger, T. G., Copeland, R. A., Lewis, B. R., and Gibson, S. T. (2003). Two-photon REMPI spectra from $a^1\Delta_g$ and $b^1\Sigma_g^+$ to $d^1\Pi_g$ in O_2. *J. Molec. Spec. 219*, 200–216.

(*4*) O'Keeffe, P., Ridley, T., Sheard, H. A., Lawley, K. P., Donovan, R. J., and Lewis, B. R. (2002). The $d^1\Pi_g(v = 1)$ Rydberg state of O_2: Optical-optical double-resonance and Huggins-band ozone-photolysis, resonance-enhanced multiphoton-ionization studies with a $b^1\Sigma_g^+(v = 0)$-state platform. *J. Chem. Phys. 117*, 8705–8709.

(*5*) Warter, M. L., Gunthardt, C. E., Wei, W., McBane, G. C., and North, S. W. (2018). Nascent $O_2(a^1\Delta_g, v = 0, 1)$ rotational distributions from the photodissociation of jet-cooled O_3 in the Hartley band. *J. Chem. Phys. 149*, 134309.

(*6*) Gunthardt, C. E., Aardema, M. N., Hall, G. E., and North, S. W. (2019). Evidence for lambda doublet propensity in the UV photodissociation of ozone. *J. Chem. Phys. 151*, 224302.

(*7*) Ulrich, C. K., Chen, J., Tokel, O., Houston, P. L., and Grebenshchikov, S. Y. (2013). Photodissociation of Ozone from 321 to 329 nm: The Relative Yields of $O(^3P_2)$ with $O_2(X^3\Sigma_g^-)$, $O_2(a^1\Delta_g)$ and $O_2(b^1\Sigma_g^+)$. *J. Phys. Chem. A 117*, 12011–12019.

(*8*) Morrill, J. S., Ginter, M. L., Lewis, B. R., and Gibson, S. T. (1999). The $(X^2\Pi_g)ns\sigma_g^{1,3}\Pi_g$ Rydberg states of O_2: Spectra, structures, and interactions. *J. Chem. Phys. 111*, 173–185.

(*9*) van der Zande, W. J., Koot, W., Los, J., and Peterson, J. R. (1988). Predissociation of the $d^1\Pi_g$ Rydberg state in $\mathrm{O_2}$: Nature of the Rydberg-valence interactions. *J. Chem. Phys. 89*, 6758–6770.

(*10*) van der Zande, W. J., Koot, W., Peterson, J. R., and Los, J. (1987). Charge Exchange of $\mathrm{O_2^+}$ with Cs: Spectroscopy and Predissociation Pathways for the Π_g Rydberg States of $\mathrm{O_2}$. *Chem. Phys. Lett. 140*, 175–180.

(*11*) Le Roy, R. J., and Kraemer, G. T. (2004). BCONT 2.2 A Computer Program for Calculating Bound $\rightarrow$ Continuum Transition Intensities for Diatomic Molecules. *University of Waterloo Chemical Physics Research Report CP-650R^2*.

(*12*) Li, Y., Petsalakis, I. D., Liebermann, H.-P., Hirsch, G., and Buenker, R. J. (1997). Ab initio configuration interaction calculations of the predissociation of rovibrational levels of the $C^3\Pi_g$ and $d^1\Pi_g 3s\sigma$ Rydberg states of the oxygen molecule. *J. Chem. Phys. 106*, 1123–1133.

(*13*) Bray, R. G., and Hochstrasser, R. M. (1976). Two-photon absorption by rotating diatomic molecules. *Molec. Phys. 31*, 1199–1211.

(*14*) O'Keeffe, P., Ridley, T., Lawley, K. P., Maier, R. R. J., and Donovan, R. J. (1999). Kinetic energy analysis of $O(^3P)$ and $O_2(b^1\Sigma_g^+)$ fragments produced by photolysis of ozone in the Huggins bands. *J. Chem. Phys. 110*, 10803–10809.

(*15*) O'Keeffe, P., Ridley, T., Lawley, K. P., and Donovan, R. J. (2001). Re-analysis of the ultraviolet absorption spectrum of ozone. *J. Chem. Phys. 115*, 9311–9319.
